# Annexin A1 restores cerebrovascular integrity concomitant with reduced amyloid-β and tau pathology

**DOI:** 10.1093/brain/awab050

**Published:** 2021-06-21

**Authors:** Miriam Ries, Helena Watts, Bibiana C Mota, Maria Yanez Lopez, Cornelius K Donat, Nicoleta Baxan, James A Pickering, Tsz Wing Chau, Annika Semmler, Brinda Gurung, Robertas Aleksynas, Laura Abelleira-Hervas, Soha Jamshed Iqbal, Carmen Romero-Molina, Gerard Hernandez-Mir, Antonio d’Amati, Chris Reutelingsperger, Marc H Goldfinger, Steve M Gentleman, Fred Van Leuven, Egle Solito, Magdalena Sastre

**Affiliations:** 1 Department of Brain Sciences, Imperial College London, London, UK; 2 Biological Imaging Centre, Imperial College London, London, UK; 3 William Harvey Research Institute, Queen Mary University London SMD, London, UK; 4 Department of Biochemistry, Cardiovascular Research Institute Maastricht, Maastricht University, Maastricht, The Netherlands; 5 Experimental Genetics Group-LEGTEGG, Department of Human Genetics, KU Leuven, Leuven, Belgium; 6 Dipartimento di Medicina Molecolare e Biotecnologie Mediche, Universitá degli Studi di Napoli “Federico II”, Naples, Italy

**Keywords:** Aβ, BBB, ANXA1, IDE, tau

## Abstract

Alzheimer’s disease, characterized by brain deposits of amyloid-β plaques and neurofibrillary tangles, is also linked to neurovascular dysfunction and blood–brain barrier breakdown, affecting the passage of substances into and out of the brain. We hypothesized that treatment of neurovascular alterations could be beneficial in Alzheimer’s disease. Annexin A1 (ANXA1) is a mediator of glucocorticoid anti-inflammatory action that can suppress microglial activation and reduce blood–brain barrier leakage. We have reported recently that treatment with recombinant human ANXA1 (hrANXA1) reduced amyloid-β levels by increased degradation in neuroblastoma cells and phagocytosis by microglia. Here, we show the beneficial effects of hrANXA1 *in vivo* by restoring efficient blood–brain barrier function and decreasing amyloid-β and tau pathology in 5xFAD mice and Tau-P301L mice. We demonstrate that young 5xFAD mice already suffer cerebrovascular damage, while acute pre-administration of hrANXA1 rescued the vascular defects. Interestingly, the ameliorated blood–brain barrier permeability in young 5xFAD mice by hrANXA1 correlated with reduced brain amyloid-β load, due to increased clearance and degradation of amyloid-β by insulin degrading enzyme (IDE). The systemic anti-inflammatory properties of hrANXA1 were also observed in 5xFAD mice, increasing IL-10 and reducing TNF-α expression. Additionally, the prolonged treatment with hrANXA1 reduced the memory deficits and increased synaptic density in young 5xFAD mice. Similarly, in Tau-P301L mice, acute hrANXA1 administration restored vascular architecture integrity, affecting the distribution of tight junctions, and reduced tau phosphorylation. The combined data support the hypothesis that blood–brain barrier breakdown early in Alzheimer’s disease can be restored by hrANXA1 as a potential therapeutic approach.

## Teaser

Dysfunction of the blood–brain barrier is increasingly accepted as an early biomarker for Alzheimer’s disease. Ries *et al.* show that the acute administration of the anti-inflammatory protein annexin A1 (ANXA1) reverses vascular damage while also reducing amyloid and tau pathology in mouse models of the disorder.

## Introduction

Alzheimer’s disease, the most common form of dementia, is a complex disorder characterized by the progressive decline in memory and cognition, and the accumulation of amyloid-β and tau in the brain, associated with extensive neuroinflammation.^1^ Vascular disruption is evident in Alzheimer’s disease brain^2^ with blood–brain barrier leakage even at early stages.^3^ Damaged blood vessels and blood–brain barrier breakdown were initially associated with amyloid-β and tau pathology in animal models.^4^^,^^5^ More recently, these alterations were observed in individuals with early cognitive dysfunction regardless of amyloid-β or tau markers.^6^ Therefore, blood–brain barrier disruption is becoming accepted as an early biomarker for Alzheimer’s disease.^7–10^ It remains to be determined whether this is a consequence of neuroinflammation or amyloid-β or tau pathology.

Additionally, cerebrovascular alterations can affect the progression of Alzheimer’s disease. The original proposal that disturbed cerebral microvascular function could contribute to the pathogenesis of Alzheimer’s disease^8^ still remains, more than a quarter of a century later, a matter of controversy. Vascular alterations may only be a risk factor rather than disease initiating.^11^ The rationale to link Alzheimer’s disease to blood–brain barrier leakage is evidently the failure of normal control of passage of substances into the brain.^12^ Even cells like peripheral circulating leucocytes might move across the blood–brain barrier, increasing central neuroinflammation. The release of IFN-γ from infiltrating Th1 cells significantly accelerated disease in an animal model of Alzheimer’s disease.^13^ Increased inflammation in the brain is associated with neuronal damage and accelerated amyloid-β generation.^14^ Therefore, initial breakdown of the blood–brain barrier could lead to further vascular damage and Alzheimer’s disease pathology by affecting the levels of inflammatory mediators and the production of amyloid-β. The combined evidence suggests that targeting vascular damage is a potential avenue to treat Alzheimer’s disease.

Annexin A1 (ANXA1) is a mediator of the anti-inflammatory actions of glucocorticoids, which has been largely described in the peripheral nervous system.^15^ In addition to its known role as a pro-resolving mediator in peripheral inflammation,^16^ it also modulates microglial activation.^17^ ANXA1 expression in microglia is essential for phagocytosis of apoptotic cells.^17^^,^^18^ In addition, ANXA1 inhibits neutrophil infiltration and decreases inflammatory responses,^19^ while its deletion resulted in exacerbation of the inflammatory response.^20^^,^^21^ Besides its anti-inflammatory properties, we showed that ANXA1 controls blood–brain barrier permeability.^22^*In vitro* and *in vivo* experiments have demonstrated that ANXA1 regulates blood–brain barrier integrity in brain endothelial cells and can restore functionality in ANXA1 knockout mice.^22^ The effect of ANXA1 on the blood–brain barrier appears to be exerted via activation of formyl peptide receptor-2 (FPR2),^23^ which is expressed on brain microvascular endothelial cells.^22^ Particularly, ANXA1 binding to FPR2 inhibits RhoA signalling,^22^ leading to cytoskeletal stabilization, ultimately decreasing paracellular permeability.

ANXA1 further appeared as a protective agent in Alzheimer’s disease by affecting amyloid-β clearance, following our recent report that ANXA1 reduced amyloid-β levels *in vitro* by increasing both microglial uptake and its enzymatic degradation in neurons.^24^ Therefore, we hypothesized that ANXA1 could be of therapeutic use in Alzheimer’s disease by regulating blood–brain barrier leakage, reducing microvascular damage and amyloid-β pathology. In this study, we administered human recombinant ANXA1 (hrANXA1) to young 5xFAD mice, a widely accepted model of Alzheimer’s disease, resulting in the reversal of cerebrovascular disruption and the reduction in amyloid-β pathology and memory loss at an early stage of Alzheimer’s disease. In addition, we assessed the effect of acute hrANXA1 treatment in a model of tauopathy, the Tau-P301L transgenic mouse. Our results underline the potential benefit of ANXA1 in reducing blood–brain barrier leakage, as a promising treatment of Alzheimer’s disease.

## Materials and methods

### Animals

Three- and 6-month-old male 5xFAD heterozygous transgenic mice and wild-type littermates were used (JAX^®^ MMRRC Stock # 034840). Under control of the neuron-specific *Thy1* promoter, 5xFAD mice overexpress both human APP (695) with the Swedish (K670N, M671L), Florida (I716V) and London (V717I) Familial Alzheimer’s Disease (FAD) mutations, as well as human presenilin 1 containing two FAD mutations, M146L and L286V.^25^

Sixteen Tau-P301-L male mice aged 7–8 months, which express the human P301L tau mutation under the *Thy1* promoter, were provided by Fred Van Leuven.^26^

Eleven heterozygous Tau-P301S female mice aged 5–6 months, which express the human P301S mutation under the *Thy1* promoter, were provided by Michel Goedert.^27^

Mice were kept in a specific-pathogen-free facility in individually ventilated cages with a 12:12 h light: dark cycle, constant temperature and humidity, and food and water *ad libitum* unless otherwise stated. All mice were randomly assigned to treatment groups.

### Human recombinant ANXA1 treatment

For acute treatment, 4–15 mice per group, as indicated in the ‘Results’ section and figure legends, received an intravenous injection of either vehicle or of 0.67 µg/kg of hrANXA1, produced and purified as published^28^ dissolved in 0.9% saline. FITC-labelled hrANXA1 was produced as previously reported.^29^

For the subchronic treatment, 6–12 mice per group were treated daily in the morning for 1 week with 1 µg hrANXA1 (R&D Systems) intraperitoneally in 0.9% saline, or with saline vehicle.

Passage through the blood–brain barrier was estimated by intravenous injection of FITC-hrANXA1 (0.67 µg/kg) into the mice and *in vivo* imaging of their brain as detailed in the Supplementary material and Supplementary Fig. 7. In addition, we analysed endocytosis of FITC-hrANXA1 in endothelial cell cultures (Supplementary Fig. 8).

### Tissue collection

Animals were anaesthetized with an overdose of sodium pentobarbital (intraperitoneally), right ventricular blood was collected, and mice were transcardially perfused with ice-cold 0.9% saline. Brains were dissected out and the left hemisphere was fixed for 48 h in 4% paraformaldehyde in PBS and stored at 4°C in PBS with sodium azide. The right hemispheres were dissected, flash-frozen, and stored at −80°C until use. Right-ventricular blood was allowed to clot for 15 min at 37°C, then centrifuged at 1100*g* for 10 min at room temperature. The clear serum fraction was extracted and stored at −20°C until use.

### Evans blue assay

An Evans blue dye assay to assess blood–brain barrier permeability was carried out as previously described.^22^ Briefly, 24 h after acute hrANXA1 treatment, mice were injected with 4 ml/kg of 2% Evans blue in 0.9% saline intravenously, and after 1 h, anaesthetized with an overdose of sodium pentobarbital, right ventricular blood collected, mice transcardially perfused with 0.9% saline and brains dissected and homogenized. Brain homogenates were combined 6:5 with 60% trichloroacetic acid and serum extracted as described above. Absorbance of brain homogenates and serum at 620 nm was determined using a microplate reader (Molecular Devices), and dye concentration calculated using a standard curve. Brain dye content was normalized to homogenized tissue mass and serum dye content.

### Determination of amyloid-β and APP C-terminal domains

Amyloid-β, full length APP, and β-C-terminus fragments were measured by western blot analysis using 4–12% NuPAGE^TM^ gels (Invitrogen) followed by transfer to nitrocellulose membranes and immunodetection with antibody 6E10 as described previously.^30^

### Quantitative PCR

RNA was extracted from brain tissue as previously described.^31^ Levels of mRNA were analysed using a two-step method as described previously with a Stratagene Mx3000p block cycler, and 2^−ΔΔCT^ was calculated relative to *Gapdh*.^76^ Primers used are listed in Table 1.

**Table 1:** Quantitative RT-PCR primers

Gene	Forward 5′→3′	Reverse 5′→3′
*Arg1*	CAGCACTGAGGAAAGCTGGT	CAGACCGTGGGTTCTTCACA
*Cdh5*	CACTGCTTTGGGAGCCTTC	GGGGCAGCGATTCATTTTTCT
*Cldn5*	GCAAGGTGTATGAATCTGTGCT	GTCAAGGTAACAAAGAGTGCCA
*Gapdh*	ACCACAGTCCATGCCATCAC	TCCACCACCCTGTTGCTGTA
*Il1b*	TGCCACCTTTTGACAGTGATG	AAGGTCCACGGGAAAGACAC
*Il4*	GGTCTCAACCCCCAGCTAGT	GCCGATGATCTCTCTCAAGTGAT
*Il6*	ATGGATGCTACCAAACTGGAT	TGAAGGACTCTGGCTTTGTCT
*Nos2*	TGGTGAAGGGACTGAGCTGT	CTGAGAACAGCACAAGGGGT
*Ocln*	TTGAAAGTCCACCTCCTTACAGA	CCGGATAAAAAGAGTACGCTGG
*Tgfb1*	GGATACCAACTATTGCTTCAGCTCC	AGGCTCCAAATATAGGGGCAGGGTC
*Tjp1*	GGAGCTACGCTTGCCACACT	GGTCAATCAGGACAGAAACACAGT
*Tnf*	AGGGATGAGAAGTTCCCAAATG	CACTTGGTGGTTTGCTACGAC
*Vegfa*	GCACATAGAGAGAATGAGCTTCC	CTCCGCTCTGAACAAGGCT

### Immunohistochemistry and immunofluorescence

Fixed brain hemispheres were cut into 40 µm sagittal sections with a vibratome (Leica). Sections were incubated with primary antibodies and were incubated overnight at 4°C, including anti-Aβ MOAB-2 (6C3) (Millipore) at 1:1000; and anti-GFAP (Invitrogen) at 1:500, anti-Iba1 (Wako) at 1:500; anti-synaptophysin (Santa Cruz) at 1:200, anti-pan-laminin (Sigma) at 1:300; anti-fibrinogen (Dako) at 1:2000; anti-AQP4 (Santa Cruz) at 1:100 or 1:200; anti-occludin (Invitrogen) at 1:100 in 2% normal horse serum in Tris-buffered saline (TBS)-Triton 0.02%. The following day, sections were incubated with appropriate secondary antibodies (1:400; Vector), followed by enhancement with avidin-biotin complex (ABC; Vector) and 3,3′-diaminobenzidine (DAB; Sigma). Images were acquired with a Leica DM2500 microscope connected to a camera (Q-Imaging Micropublisher 3.3 RTV) using Q-capture Pro software. For immunofluorescence, fluorescent secondary antibodies (1:400 Alexa Fluor^®^, Invitrogen) were used. In some cases, staining with 647-labelled tomato lectin (Vector Labs) was carried out simultaneously with secondary antibody incubation. For Thioflavin S staining, sections were incubated with 1% Thioflavin S (Sigma) for 8 min before being washed with ethanol and H_2_O.

### FASTClear

To enable the 3D visualization of brain vessels, FASTClear was carried out. Brain sections (400-µm to 1-mm thick) were delipidated, permeabilized, stained, and refractive-index matched as previously described.^32^ Fixed tissue was delipidated in 4% SDS-boric acid buffer at 50°C for a minimum of 5 days. The tissue was then washed thoroughly in PBS with 0.1% Triton^TM^ X-100 (PBS-Triton) at 50°C (3 × 1 h). Then, the tissue was permeabilized and blocked in 0.6 M glycine, 0.2% Triton^TM^ X-100, 6% donkey serum, 20% dimethyl sulfoxide (DMSO) dissolved in PBS overnight at 37°C. Next, after washing the tissue in PBS-Triton for 2 × 1 h at 37°C, it was incubated with primary antibody diluted in 0.2% Tween-20, 5% DMSO, 3% donkey serum, 0.01% sodium azide in PBS for a minimum of 2 days at 37°C. Following another wash in PBS-Triton (3 × 1 h, then overnight incubation at 37°C), the tissue was incubated with a secondary antibody conjugated with Alexa Fluor^®^ fluorophores diluted in the same diluent as above for the same number of days as primary antibody incubation at 37°C. After that, the tissue was washed thoroughly in PBS-Triton (5 × 1 h, then overnight) and proceeded to refractive-index matching. For tissues that have been rendered optically transparent at the delipidation step, immersion in 47% 2,2′-thiodiethanol (TDE) (vol/vol) in 0.01 M PBS as previously described^33^ was done for refractive-index matching.

### Statistical analysis

Data shown in graphs represent the mean value ± standard error of the mean (SEM). Statistical analysis was performed using GraphPad Prism 7 software. A value of *P < *0.05 was considered statistically significant. Significance values indicated on graphs correspond to the following: *P < 0.05, **P < 0.01, ***P < 0.001, ****P < 0.0001. A Kolmogorov-Smirnov normality test was used to determine whether data followed a Gaussian distribution. Data showing a normal distribution were analysed using an unpaired or paired two-tailed Student’s *t*-test, ordinary one-way ANOVA, or repeated measures one-way ANOVA. If ANOVA group effects were detected, *post hoc* analysis was carried out using a Bonferroni or Tukey’s multiple comparisons post-test. Data showing an abnormal distribution were analysed using the equivalent non-parametric tests, namely a two-tailed Mann-Whitney test, two-tailed Wilcoxon matched-pairs signed rank test, Kruskal-Wallis test, or Friedman test. If Kruskal-Wallis or Friedman group effects were observed, *post hoc* analysis was carried out using a Dunn’s multiple comparisons test. For correlations, linear regression analysis was used to determine Pearson’s correlation coefficient *r*. All behavioural data were analysed using the parametric tests described above.

Additional materials and methods are provided in the Supplementary material.

### Study approval

All animal procedures were approved by the UK Home Office (license number 70/7485) and were in accordance with the Animals (Scientific Procedures) Act of 1986 and European Union Directive 2010/63/EU.

### Data availability

The data that support the findings of this study are available from the corresponding author, upon reasonable request.

## Results

### Human recombinant ANXA1 reverses blood–brain barrier leakage in young 5xFAD mice

**Figure 1:**
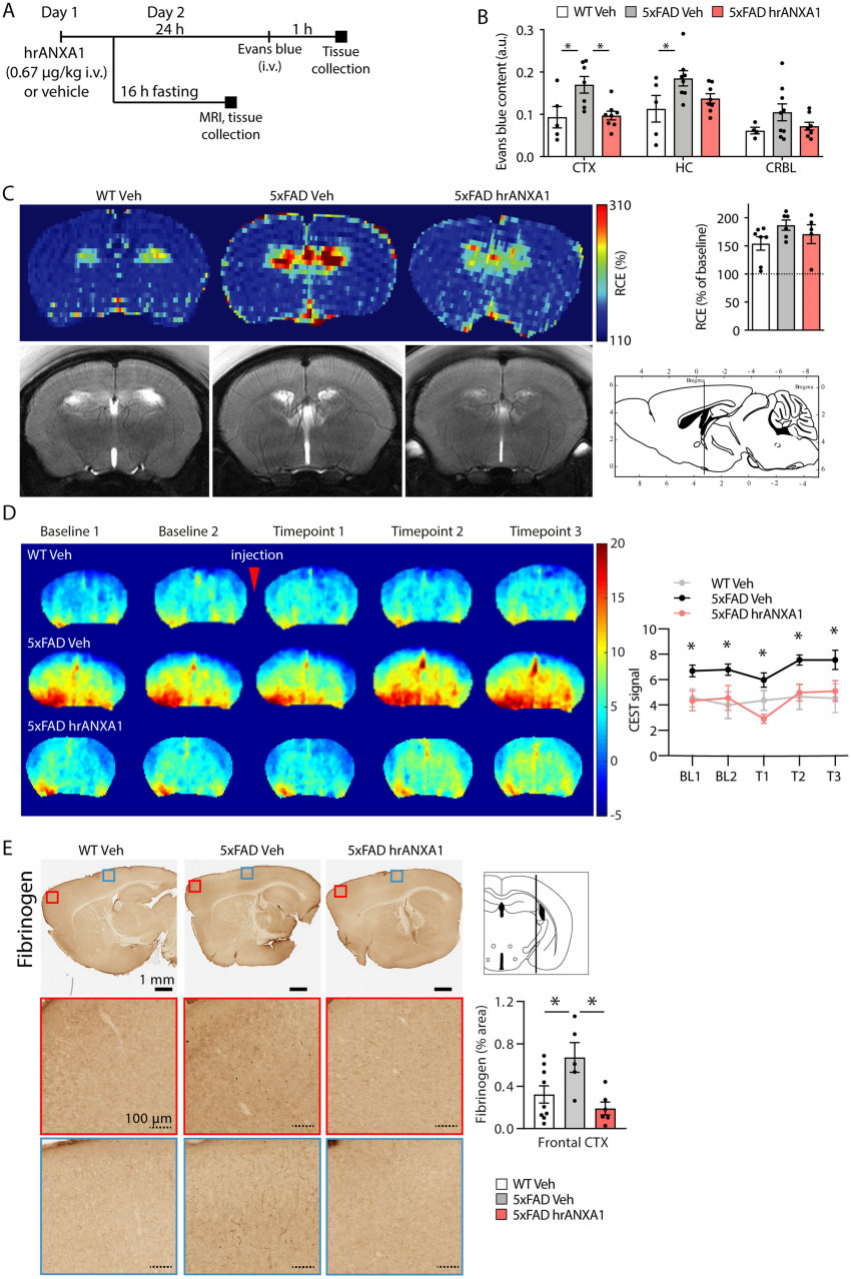
**Blood–brain barrier permeability is increased in young 5xFAD mice and rescued by hrANXA1 treatment.** (**A**) Schematic representation of acute treatment schedule of wild-type and 5xFAD mice with vehicle or hrANXA1 (0.67 µg/kg i.v.). (**B**) Quantification of Evans blue dye assay of blood–brain barrier permeability 24 h after hrANXA1 or vehicle treatment, normalized to serum dye content and brain tissue weight. Frontal cortex (CTX, *n = *5–8), hippocampus (HC, *n = *5–8), cerebellum (CRBL, *n = *4–9). One-way ANOVA with *post hoc* Bonferroni multiple comparisons test. (**C**) Representative DCE-MRI derived images and quantification of relative contrast enhancement (RCE) in wild-type (WT) mice and 5xFAD mice 24 h after hrANXA1 or vehicle treatment (*n = *5–7/group, average of two slices per mouse). T_1_-weighted multi-slice images were continuously acquired from 3 min before to 10 min after 100 μl Gd-DTPA (1 mmol/ml) intravenous injection at a rate of 0.6 ml/min. (**D**) Representative maps and quantification in the whole brain of repeated single slice CEST measurements at baseline (two measures) and at three time points after intravenous injection (0.15 ml/min) of 200 μl of 0.5 g/ml d-glucose solution in mice 24 h after hrANXA1 or vehicle treatment (*n = *4/group). Units represent percentage CEST effect (0.8–2.2 ppm maps). Multiple *t*-tests corrected for multiple comparisons, adjusted *P*-values < 0.05 between 5xFAD vehicle (Veh) and 5xFAD hrANXA1 at all time points (asterisks). (**E**) Representative staining for fibrinogen and quantification in sagittal brain sections of wild-type mice and 5xFAD mice 24 h after hrANXA1 or vehicle treatment (*n = *5–9 per group). One-way ANOVA followed by Tukey’s multiple comparisons test. Bars throughout represent mean ± SEM. *P < 0.05. Mouse brain atlas images were obtained from the Allen Institute website (www.alleninstitute.org).

The alterations in blood–brain barrier permeability and the potential effects of hrANXA1 treatment (Fig. 1A) were analysed in a mouse model of amyloidosis, first by the Evans blue dye extravasation assay in young 5xFAD mice (aged 3 months). The spectrophotometric measurement of brain dye content revealed increased blood–brain barrier permeability in the cortex and hippocampus compared to wild-type littermates (*P < *0.05) without significant changes in the cerebellum (Fig. 1B). Interestingly, acute treatment with hrANXA1 reversed this effect significantly in the cortex (*P < *0.05, Fig. 1B). At 6 months of age, no differences in blood–brain barrier leakage were observed between 5xFAD mice treated either with vehicle or ANXA1 and age-matched wild-type controls (Supplementary Fig. 3). Therefore, we focused our study on the effects of ANXA1 at the early stage of the disease.

The blood–brain barrier leakage in young 5xFAD mice and its reduction with hrANXA1 treatment was subsequently confirmed by MRI following administration of dimeglumine gadopentetate (Gd) contrast agent (Fig. 1C). The higher Gd signals in the hippocampus were consistent with blood–brain barrier leakage in young 5xFAD mice (vehicle-treated) and the effect was normalized by treatment with hrANXA1 (Fig. 1C). We then used glucose chemical exchange saturation transfer (glucoCEST) molecular MRI based on proton exchange to define specific molecules.^34^ On the same animals, we performed CEST MRI using d-glucose as an alternative/complement to dynamic contrast enhanced (DCE) imaging to examine blood–brain barrier breakdown.^35^ The results suggest an increase of d-glucose uptake in young 5xFAD mice compared to wild-type mice and to hrANXA1 treated 5xFAD mice at baseline and at all time points after d-glucose injection (Fig. 1D). The statistically significant differences between treated and untreated 5xFAD mice were observed at all time points (multiple *t*-tests corrected for multiple comparisons, adjusted *P*-values < 0.05); this suggests that the change in glucose was not related to blood–brain barrier leakage of d-glucose, since the difference was already apparent at the baseline time points, before the d-glucose was injected into the blood.

The spectroscopic results were extended by immunohistochemical visualization of fibrinogen, a major blood plasma protein, in the brain of the three groups of mice (wild-type, 5xFAD, hrANXA1-treated 5xFAD) (Fig. 1E). Quantitatively and qualitatively, extravasation of fibrinogen from blood vessels was evident in the cortical areas of young 5xFAD mice, with less staining in the wild-type mice and in the 5xFAD mice treated with hrANXA1 (Fig. 1E and Supplementary Fig. 1C).

### Human recombinant ANXA1 restores vascular disruption in young 5xFAD mice

**Figure 2:**
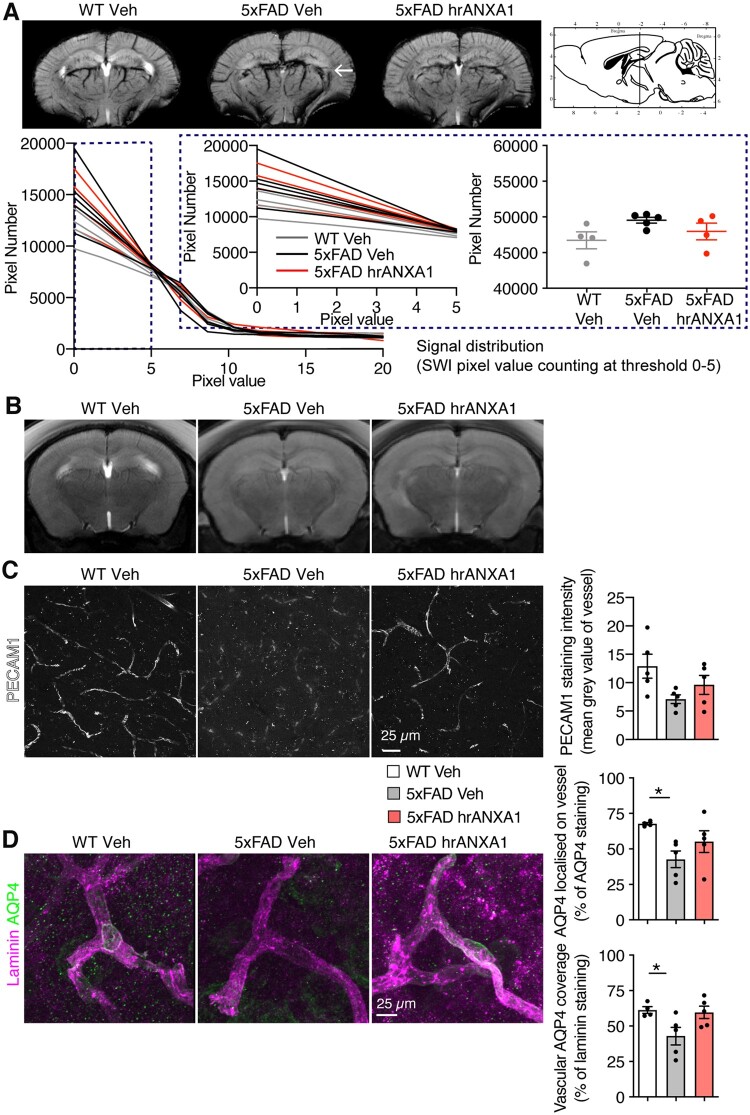
**Neurovascular impairment in young 5xFAD mice is restored by hrANXA1 treatment**. (**A**) Susceptibility-weighted MRI of wild-type and 5xFAD mouse brain 24 h after hrANXA1 or vehicle treatment showing vascular morphology. Arrow indicates microhaemorrhage. Histogram of SWI signal distribution (60 bins) ranging from 0 to 100 a.u. pixel values. Most heterogeneous distribution occurs in the interval of low pixel intensity values (highlighted region). *Right*: Denser distribution (150 bins) of the SWI signal covering an interval of pixel values from 0 to 5 a.u. (*left*). Pixel count analysis (*right*) shows a trend of increased number of low intensity pixels in vehicle 5xFAD treated mice compared to baseline (wild-type, WT). A trend of recovery to baseline values can be observed in the hrANXA1 5xFAD treated mice (*n = *4–5 per group). (**B**) T_2_-weighted morphological MRI of mice 24 h after hrANXA1 or vehicle treatment. (**C**) Representative maximum *z*-projection images and quantification of PECAM1 vascular staining intensity in the cortex (*n = *5/group, average value from 22 to 193 vessels/mouse, two to five sections stained per mouse, two images taken per section). (**D**) Representative maximum *z*-projection images and quantification of co-localization of anti-AQP4 [AQP4(4/18), white] and anti-pan-laminin (red) staining in the cortex of wild-type and 5xFAD mice 24 h after hrANXA1 or vehicle treatment. Proportion of co-localization (Mander’s overlap coefficients) in *z*-stacks was determined using ImageJ (*n = *4–5 animals per group, two sections per animal). *Bottom*: Vascular AQP4 coverage (percentage of total laminin staining co-localizing with AQP4). *Top*: AQP4 localization on vessel (percentage of total AQP4 staining co-localizing with laminin). One-way ANOVA with *post hoc* Bonferroni multiple comparisons test. Bars represent mean ± SEM. *P < 0.05. Mouse brain atlas images were obtained from the Allen Brain Atlas (www.alleninstitute.org).

The increased blood–brain barrier permeability in young 5xFAD mice was therefore studied by susceptibility-weighted imaging (SWI) to visualize alterations in brain vascular architecture. SWI revealed alterations in the vascular architecture of the 5xFAD brain already at age 3 months, including micro-haemorrhages (Fig. 2A). These defects were not evident in the young 5xFAD mice treated with hrANXA1 (Fig. 2A). No gross differences were observed in brain morphology in the three types of mice by T_2_-MRI (Fig. 2B).

To define structural alterations further, we assessed the expression and distribution of vascular markers, first by immunohistochemical staining for the established endothelial cell marker PECAM1 (CD31). The disruption of small vessel architecture became thereby evident in young 5xFAD mice, compared to age-matched wild-type littermates, in addition to the treatment with hrANXA1 that visibly improved their blood vessel morphology (Fig. 2C). The general minor effect on the intensity of the staining indicated that not the overall level of PECAM1 was affected but its actual cellular localization and thereby restoration of the anatomy of the vessels.

Next, we evaluated another important component of the neurovascular unit, the astrocyte end-feet that surround small cerebral blood vessels. The water channel AQP4 is primarily located here, and thereby a specific marker for these processes, and was even suggested to contribute to the clearance of amyloid-β.^37^ We therefore stained similar brain sections for the end-foot marker AQP4 in comparison to the cerebral microvascular endothelial cell marker lectin. Using tissue clearing to allow 3D visualization of the vasculature, we observed a reduction in AQP4 co-localization with lectin-positive vessels in the cortex in young 5xFAD mice compared to wild-type littermates (Supplementary Fig. 1A). Treatment with hrANXA1 of young 5xFAD mice restored co-localization of AQP4 with lectin, more comparable to the normal situation in wild-type mice.

The observed qualitative alterations of the astrocyte end-feet in the neurovascular unit were further assessed by co-immunohistochemical staining of AQP4 and the basal lamina marker laminin. Co-localization analysis revealed the reduction in relative coverage of laminin and AQP4 in young 5xFAD mice compared to age-matched wild-type littermates (Fig. 2D). Wild-type mice showed 61% AQP4-laminin co-positive vessels, in contrast to 45% in young 5xFAD mice (Fig. 2D). This highly significant difference is interpreted to mean that the AQP4-positive astrocytic endfeet in the young 5xFAD mice were dislocated off the basal lamina of the endothelial cells forming the vessel. Most interestingly, both AQP4 localization and vascular AQP4 coverage were restored back to 60% in young 5xFAD mice treated with a single dose of hrANXA1 (Fig. 2D). Total overall levels of AQP4 were estimated by western blot in brain homogenates and were unaltered in young 5xFAD mice (Supplementary Fig. 1B) implying that the hrANXA1-pretreatment affected the AQP4 subcellular localization and not its level of expression.

**Figure 3:**
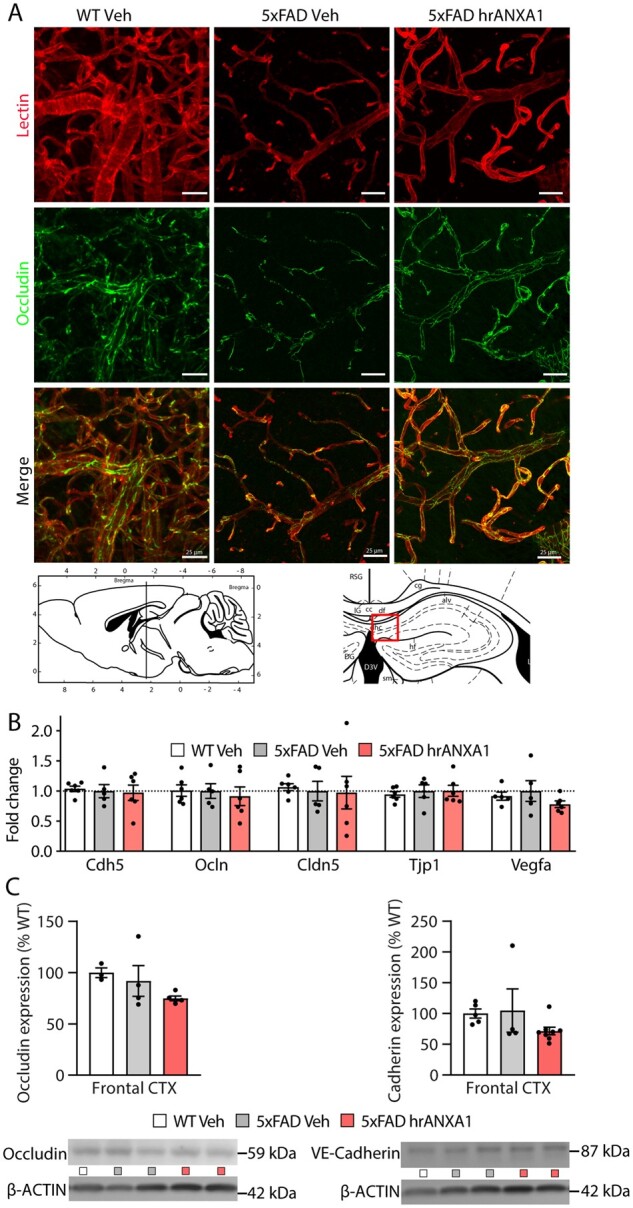
**ANXA1 affects cellular distribution of tight junctions in 5xFAD mice.** (**A**) Representative images of occludin (green) and lectin (red) staining in cortical sections that had undergone FASTClear of wild-type and 5xFAD mice treated with hrANXA1 or vehicle. (**B**) Quantification of qPCR analysis of mRNA expression of *Cdh5* (VE-cadherin), *Ocln* (occludin), *Cldn5* (Claudin 5), *Tjp1* (ZO-1), and *Vegfa* (VEGF) in the frontal cortex of wild-type and 5xFAD mouse brain treated with hrANXA1 or vehicle (*n = *5–6/group). (**C**) Quantification of protein expression of occludin and VE-Cadherin in cortical brain homogenates (*n = *3–8 per group). Bars represent mean ± SEM. Mouse brain atlas images were obtained from the Allen Institute website (www.alleninstitute.org).

We next investigated whether the alterations in cerebrovascular integrity in young 5xFAD mice were caused by changes in tight junctions. Brain sections from age-matched young wild-type and 5xFAD mice were stained for the tight junction marker occludin and with the cerebrovascular marker tomato lectin. Subsequent tissue clearing allowed the 3D visualization of the vascular unit (Fig. 3A). The co-localization of occludin with tomato lectin was visibly reduced in the cortex of young 5xFAD mice compared to wild-type littermates, pointing to the loss of tight junctions (Fig. 3A). Moreover, in the brain of young 5xFAD mice that were acutely treated with hrANXA1, the pattern of occludin and tomato lectin co-staining was very similar to that in wild-type mice (Fig. 3A). This outcome *in vivo* is completely in line with previous cell-based results, showing that hrANXA1 decreased blood–brain barrier leakage by affecting the distribution of membrane-associated tight junctions in human microvascular endothelial cells.^22^

We ruled out that the observed changes in occludin distribution by treatment with hrANXA1 in young 5xFAD mice were caused by transcriptional expression of intercellular junction components. Indeed, quantitative PCR analysis and total protein expression of different markers of tight and adherens junctions demonstrated no alterations in the levels of *Ocln* nor of other junction markers: VE-cadherin (cadherin 5, *Cdh5*), claudin 5 (*Cldn5*), ZO-1 (tight junction protein 1, *Tjp1*) nor of the growth factor VEGF-A (*Vegfa*), in young 5xFAD mice, whether treated with hrANXA1 or vehicle (Fig. 3B and C).

The combined datasets demonstrate that hrANXA1 restored the vascular endothelial damage in young 5xFAD mice by affecting not the expression, but the distribution of specific proteins essentially involved in tight junctions and astrocytic endfeet in the brain vascular unit.

### Acute administration of ANXA1 affects amyloid-β clearance

**Figure 4:**
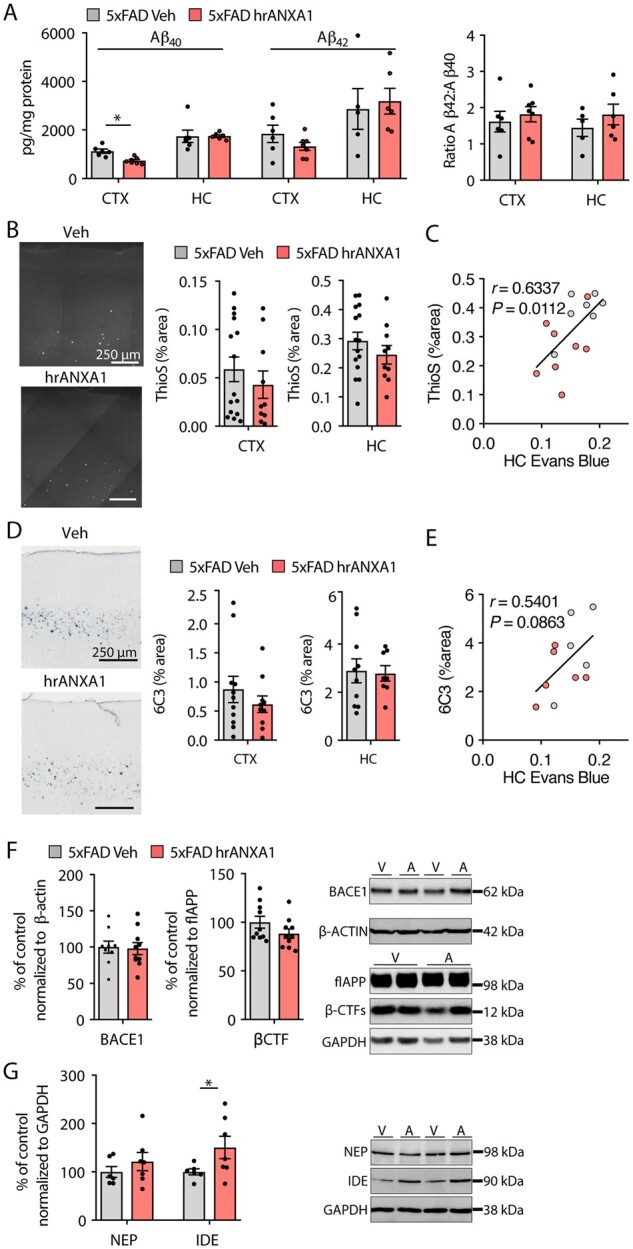
**Acute hrANXA1 treatment reduces cortical amyloid-β_40_ pathology by increasing amyloid-β degradation in 5xFAD mice.** (**A**) *Left*: ELISA analysis of amyloid-β_40_ and amyloid-β_42_ in the motor cortex (CTX) and hippocampus (HC) of 5xFAD mouse brain treated with hrANXA1 or vehicle, expressed as picograms per milligrams of protein (*n = *6–7/group). *Right*: Ratio of amyloid-β_42_ and amyloid-β_40_ detected by ELISA in the motor cortex and hippocampus. Unpaired two-tailed *t*-test. (**B**) Representative images and quantification of percentage area of Thioflavin-S staining in the cortex (CTX) and hippocampus (HC) of 5xFAD mice treated with hrANXA1 or vehicle (*n = *10–15 mice/group, mean of four to six sections analysed per mouse). (**C**) Scatter plot showing significant positive correlation between Evans blue dye content and Thioflavin-S staining percentage area in the hippocampus of 5xFAD mice acutely treated with 0.67 µg/kg hrANXA1 or vehicle at 3 months of age (*n = *7–8/group, linear regression analysis, Pearson’s *r* = 0.6337, *P *=* *0.0112 (vehicle and ANXA1-treated). (**D**) Representative images and quantification of percentage area of anti-amyloid-β (6C3 antibody) in the cortex and hippocampus of 5xFAD mice treated with hrANXA1 or vehicle (*n = *8–11 mice/group, mean of four to six sections analysed per mouse). (**E**) Scatter plot showing significant positive correlation between Evans blue dye content and 6C3 percentage area stained in the hippocampus of 5xFAD mice acutely treated with 0.67 µg/kg hrANXA1 or vehicle at 3 months of age (*n = *5–6/group, linear regression analysis, Pearson’s *r* = 0.5401, *P *=* *0.086). (**F**) Representative western blots and quantitative analysis of β-secretase expression and β-CTFs levels in 5xFAD mice treated with hrANXA1 or vehicle. BACE1 expression in the motor cortex, normalized to β-actin (*n = *9–10/group). β-CTF expression in the motor cortex, normalized to flAPP (*n = *10/group). (**G**) Representative western blots and quantitative analysis of neprilysin (*n = *6–7/group) and IDE expression (*n = *6/group) in the motor cortex of 5xFAD mice treated with hrANXA1 or vehicle, normalized to GAPDH. Unpaired two-tailed *t*-test. Columns represent mean ± SEM. *P < 0.05.

To investigate whether hrANXA1 treatment affected amyloid-β metabolism and deposition *in vivo*, we first assessed by ELISA the amyloid-β levels in brain homogenates from young 5xFAD mice treated with hrANXA1 or with vehicle. The small but significant reduction in amyloid-β_40_ was evident in the cortex of mice treated with hrANXA1 compared with vehicle-treated control 5xFAD mice (*P < *0.05) (Fig. 4A). No significant changes were observed in the levels of amyloid-β_42_ or in the ratio of both forms (Fig. 4A). The amyloid-β load and plaque burden in cortex and hippocampus estimated by immunohistochemistry were not significantly affected by hrANXA1 (Fig. 4B and D). Interestingly, strong positive correlations were observed between the values of amyloid-β plaque load estimated by Thioflavin-S staining and the blood–brain barrier permeability estimated by Evans blue extravasation in the hippocampus (Fig. 4C, linear regression analysis, Pearson’s *r *=* *0.6337, *P *=* *0.0112), but were not significant in the case of total amyloid-β load and the blood–brain barrier permeability (Fig. 4E, linear regression analysis, Pearson’s *r *=* *0.540, *P *=* *0.086) in both vehicle and ANXA1-treated 5xFAD mice. These data suggest the association of high blood–brain barrier leakage and brain amyloid-β levels.

We next determined whether the reduction in amyloid-β_40_ levels was related to changes in the processing of APP. Analysis of total full-length APP and of the metabolic carboxy-terminus fragments (CTFs) as well as of BACE1, the rate-limiting enzyme in amyloid-β production, did not reveal any differences between hrANXA1-pre-treated and untreated young 5xFAD mice (Fig. 4F and Supplementary Fig. 2A), suggesting that acute hrANXA1 treatment did not affect amyloid-β generation. In contrast, increased cortical expression of the amyloid-β-degrading enzymes neprilysin and particularly IDE demonstrated that hrANXA1 acutely affected these amyloid-β degradation pathways (Fig. 4G, Supplementary Fig. 2B and C) in agreement with our previous results *in vitro.*^24^ We did not detect changes in the levels of the known clearance factors APOE and LRP1 (Supplementary Fig. 2D and E).

ANXA1 administration did not affect amyloid-β pathology or enzymatic expression in 6-month-old 5xFAD mice (Supplementary Fig. 3), suggesting that perhaps at this stage it is too late for ANXA1 to affect the removal of large amyloid deposits.

### Human recombinant ANXA1 reduced inflammatory markers and T-cell infiltration into the brain

The anti-inflammatory protein ANXA1 has been shown to resolve inflammation systemically in a number of experimental models.^16^ In addition, we recently reported that hrANXA1 inhibited the amyloid-β-stimulated secretion of inflammatory mediators in a microglia cell line.^24^ To extend and confirm these results *in vivo*, we analysed the effects of acute hrANXA1 treatment in young 5xFAD mice on several inflammatory markers.

**Figure 5:**
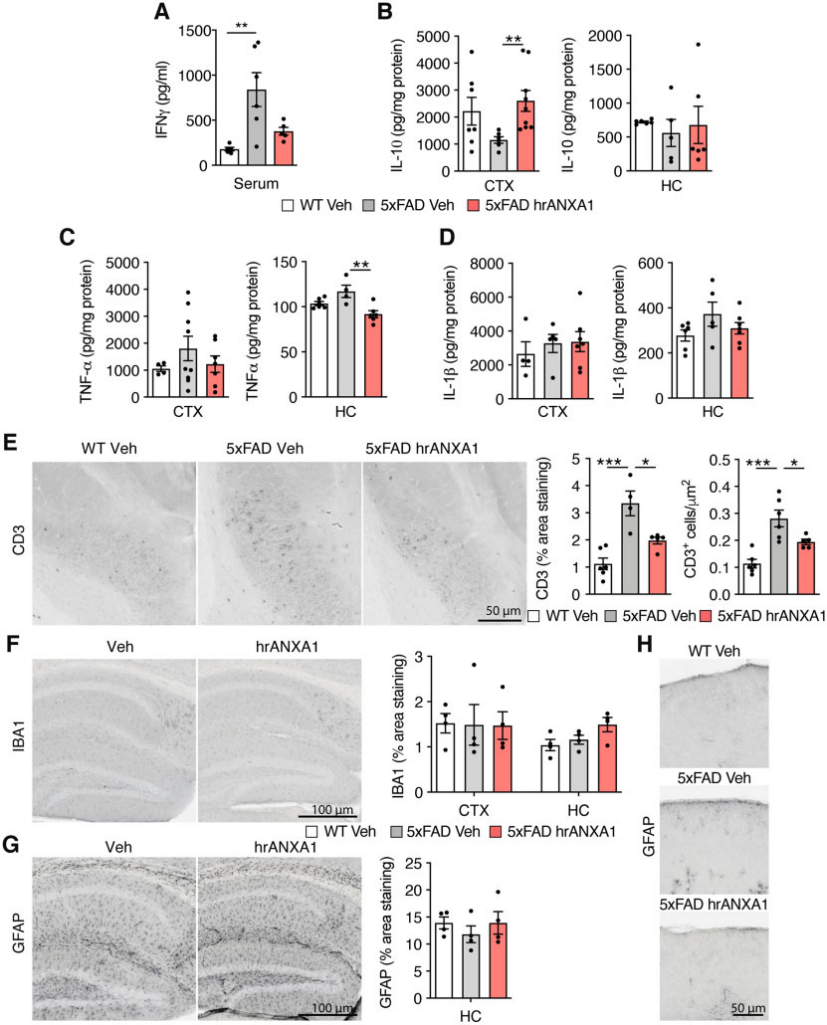
**Treatment with hrANXA1 reduced inflammatory markers and T-cell infiltration in brains of 5xFAD mice.** (**A**) ELISA analysis of IFNγ expression in the serum of wild-type and 5xFAD mice treated with hrANXA1 or vehicle (*n = *5-6). Kruskal-Wallis test with *post hoc* Dunn’s multiple comparisons test. (**B**) ELISA analysis of IL-10 in the cortex (CTX) (*n = *6–9) and hippocampus (HC) (*n = *5–6) of 5xFAD mice treated with hrANXA1 or vehicle. One-way ANOVA with *post hoc* Bonferroni multiple comparisons test. (**C**) ELISA analysis of TNFα in the cortex (CTX) (*n = *4–9) and hippocampus (HC) (*n = *4–6) of 5xFAD mice treated with hrANXA1 or vehicle. One-way ANOVA with *post hoc* Bonferroni multiple comparisons test. (**D**) ELISA analysis of IL-1β in the cortex (CTX) (*n = *4–7) and hippocampus (HC) (*n = *5–7) of 5xFAD mice treated with hrANXA1 or vehicle. One-way ANOVA with *post hoc* Bonferroni multiple comparisons test. (**E**) Representative images and quantification of percentage area covered of anti-CD3 staining (*n = *4–6 mice/group, mean of one to three sections analysed per mouse) and CD3^+^ cell/µm^2^ (*n *=* *5–6 mice/group, mean of one to three sections analysed per mouse) in the subiculum of the hippocampus of wild-type and 5xFAD mice treated with hrANXA1 or vehicle. Kruskal-Wallis test with *post hoc* Dunn’s multiple comparisons test, or one-way ANOVA with *post hoc* Bonferroni multiple comparisons test. (**F**) Representative images and quantification of the percentage area of anti-Iba1 staining in the cortex and hippocampus of wild-type and 5xFAD mice treated with hrANXA1 or vehicle (*n = *4 mice/group, mean of three to six sections analysed per mouse). (**G**) Representative images and quantification of the percentage area of anti-GFAP staining in the hippocampus of wild-type and 5xFAD mice treated with hrANXA1 or vehicle (*n = *4 mice/group, mean of five to seven sections analysed per mouse). (**H**) Representative images of anti-GFAP staining in the cortex of wild-type and 5xFAD mice treated with hrANXA1 or vehicle. Bars represent mean ± SEM. *P < 0.05, **P < 0.01, ***P < 0.001.

A 55% reduction in the concentration of the pro-inflammatory cytokine IFNγ was evident in the serum of young 5xFAD mice treated with ANXA1 compared to vehicle-treated 5xFAD mice (Fig. 5A). In the brain, hrANXA1 treatment induced a 125% increase in the expression of the anti-inflammatory cytokine IL-10 in the cortex, but not in the hippocampus (Fig. 5B). In addition, the levels of the pro-inflammatory cytokines TNFα and IL-1β were reduced after hrANXA1 treatment (Fig. 5C and D). Transcriptionally, the mRNA levels of inflammatory mediators assessed remained unchanged in the brain of treated versus untreated 5xFAD mice (Supplementary Fig. 4A).

We next wondered whether hrANXA1 treatment affected the infiltration of peripheral immune cells into the CNS. We investigated CD3+ T-cells in the brain of wild-type and 5xFAD mice acutely treated with hrANXA1 or vehicle, focusing on the subiculum, where amyloid-β plaque deposition starts in this model. A significant increase in the number of CD3+ cells was evident in the subiculum of 5xFAD mice compared to wild-type littermates (*P < *0.001). Importantly, this T-cell infiltration was significantly reduced by hrANXA1 treatment (Fig. 5E).

In contrast, we did not observe significant changes in the parameters of microglia and astroglia, after staining respectively with Iba1 and GFAP antibodies, in neither cortex nor hippocampus of young 5xFAD mice treated with hrANXA1 compared to vehicle treated mice (Fig. 5F–H). Because at this young age the density of GFAP positive cortical astrocytes was low and close to the detection limit of immunohistochemistry (Fig. 5H), we also determined the protein levels of Iba1 and GFAP by western blotting in brain homogenates. These experiments did not reveal significant effects of the treatment with hrANXA1 (Supplementary Fig. 4B and C). Finally, to determine whether microglial and astrocytic activation was affected locally, we quantified the percentage area coverage of microglia and astrocytes surrounding amyloid-β plaques. No marked differences in stained area for Iba1 and GFAP surrounding plaques was observed, neither in microglial nor in astrocytic morphology, between hrANXA1 and vehicle-treated young 5xFAD mice (Supplementary Fig. 4D).

The combined results allowed us to conclude that acute treatment with hrANXA1 did not affect glial density but affected the expression of certain neuroinflammatory markers in the brain of young 5xFAD mice.

### Human recombinant ANXA1 treatment of young 5xFAD mice reversed memory deficits

**Figure 6:**
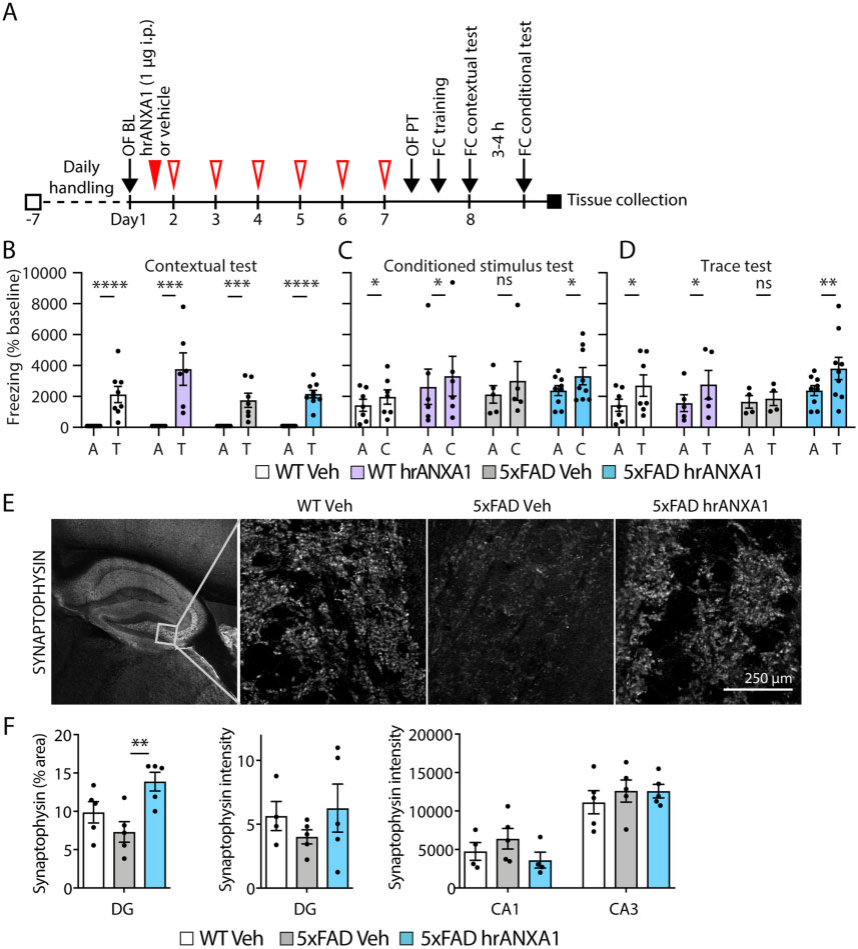
**Subchronic hrANXA1 treatment improves hippocampal-dependent memory in 5xFAD mice.** (**A**) Schematic representation of subchronic treatment schedule of wild-type and 5xFAD mice with vehicle or hrANXA1 (1 µg/day i.p. for 7 days, red arrows) and behavioural testing (black arrows). (**B**) Freezing in context in which CS-US pair training was carried out, indicative of contextual memory (*n = *6–9/group). A = acclimatization time before fear condition training; T = contextual testing. Ratio paired two-tailed *t*-test. (**C**) Freezing in novel context during acclimatization time of conditional test (A) and CS presentation (C), *n = *5–9/group. Ratio paired two-tailed *t*-test. (**D**) Freezing in novel context during acclimatization time of conditional test (A) and trace interval (T), *n = *5–9/group. Ratio paired two-tailed *t*-test. (**E**) Representative maximum *z-*projections of synaptophysin staining in the hilus of the dentate gyrus of wild-type and 5xFAD mice treated with hrANXA1 or vehicle. (**F**) Quantification of the percentage area of synaptophysin staining in the hilus of the dentate gyrus (DG) (*n = *5 mice/group, mean of two sections analysed per mouse) and synaptophysin staining intensity in the dentate gyrus and CA1 and CA3 regions of the hippocampus (*n = *5 mice/group, mean of two to five sections analysed per mouse) of wild-type and 5xFAD mice treated with hrANXA1 or vehicle. One-way ANOVA with Bonferroni multiple comparisons test. Bars represent mean ± SEM. *P < 0.05, **P < 0.01, ***P < 0.001, ****P < 0.0001

Next, we determined effects of hrANXA1 on brain structural morphology, synaptic density and behaviour in young 5xFAD mice. To assess changes in behaviour and memory, we treated mice subchronically with hrANXA1 (1 µg/day intraperitoneally for 7 days; Fig. 6A). In the fear-conditioning paradigm, a conditioned stimulus (tone) was paired with an unconditioned stimulus (mild foot shock) to assess hippocampal-dependent and hippocampal-independent learning and memory (Supplementary Fig. 5A–D). Age-matched young wild-type and 5xFAD mice treated with vehicle or with hrANXA1 showed both intact hippocampal-independent (Supplementary Fig. 5E) and hippocampal-dependent (Supplementary Fig. 5F) learning during training. Hippocampal-dependent learning in 5xFAD appeared worse than in wild-type, since the degree of freezing did not increase significantly until the trace interval after the stimulus was presented for the fourth time, while in wild-type mice this already occurred after the third stimulus presentation. 5xFAD mice pretreated with hrANXA1 also showed significant hippocampal-dependent learning after the third stimulus presentation, suggesting an improvement in learning in 5xFAD mice after hrANXA1 treatment. The freezing increased with progressive cycles of conditioned stimulation, as well as during the trace interval following the conditioned stimulus.

To evaluate context-dependent memory, mice were returned the following morning to the same environment in which they previously experienced tone-shock pairings during the training phase, and freezing was again quantified. The significant increase in freezing compared to the acclimatization before fear conditioning training observed in all mice proved an intact frontal/cingulate cortex-dependent memory (Fig. 6B).

The conditioned response was assessed in the afternoon of the day following training by placing mice in a novel context and presenting them with the same conditioned stimulus. In wild-type mice, the level of freezing in response to the conditioned stimulus in the novel context was significantly higher compared to the acclimatization time, indicative of their effective hippocampal-independent memory (Fig. 6C), and remained high after ANXA1 treatment. As expected, similarly tested vehicle-treated 5xFAD mice did not freeze significantly more after tone presentation, indicating that their hippocampal-independent memory was impaired. Most importantly, this memory defect was rescued by hrANXA1 treatment of the young 5xFAD mice (Fig. 6C).

Furthermore, hippocampal-dependent memory, analysed in the trace interval after tone presentation in the novel context, was impaired in untreated young 5xFAD, which was not the case in hrANXA1-treated 5xFAD mice, suggesting that hippocampal-dependent memory was also improved with ANXA1 treatment (Fig. 6D).

To determine whether neuronal or synaptic density correlated with the observed memory defects we undertook immunohistochemical analysis of brain slices. Cresyl violet staining to visualize neurons did not reveal significant differences between treated and untreated young 5xFAD mice, neither in the cortex (one-way ANOVA, F = 3.832, *P *=* *0.0517) nor in the subiculum (one-way ANOVA, F = 0.9698, *P *=* *0.4070, data not shown). Immunofluorescence analysis for the synaptic marker synaptophysin on hippocampal sections of the same mice revealed a significant increase in the area of synaptophysin staining in the dentate gyrus after hrANXA1 treatment of young 5xFAD mice (Fig. 6E and F).

In conclusion, the combined results underscore that subchronic administration of hrANXA1 restored memory deficits in young 5xFAD mice, which correlated with increased synaptophysin marked synaptic density in the dentate gyrus of the hippocampus.

### Effects of human recombinant ANXA1 administration in the Tau-P301L model

To investigate whether hrANXA1 also showed a protective effect in other models of Alzheimer’s disease, we tested its effectiveness at reducing tau pathology and vascular abnormalities in the Tau-P301L model of tauopathy.

**Figure 7:**
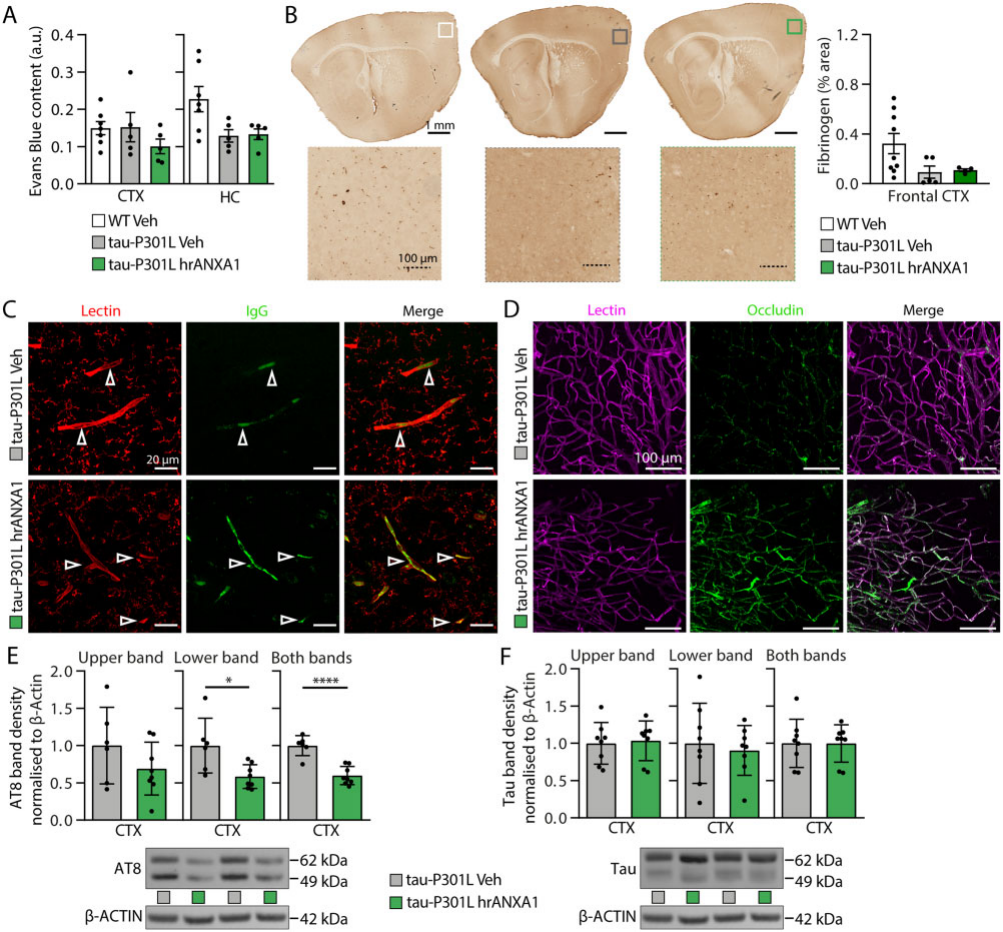
**Treatment with hrANXA1 reduces tau phosphorylation and vascular disruption in Tau-P301L mice**. (**A**) Brain Evans blue dye content measured in the frontal cortex and hippocampus of 7–8-month-old wild-type and tau P301L mice injected with Evans blue dye intravenously 24 h after acute intravenous treatment with hrANXA1 or vehicle. *n = *5–7/group. One-way ANOVA. (**B**) Representative staining for fibrinogen and quantification in sagittal brain sections of wild-type (WT) mice and Tau-P301L mice 24 h after hrANXA1 or vehicle (Veh) treatment (*n = *4–9 per group). (**C**) Representative confocal images shown as maximum *z*-projection images using ImageJ. All images are from an area of the hippocampus of wild-type, transgenic Tau-P301L and hrANXA1-treated Tau mice brains and were obtained with 63× oil objective using ZenPro. Scale bar = 20 µm. Figure shows immunofluorescence staining of endogenous IgG (green) and blood vessels with tomato lectin (red). (**D**) *Z*-stack FASTClear representative images showing staining of the vasculature in a Tau-P301L mouse. The *top row* displays results from a vehicle treated mouse, while the *bottom row* shows results from a mouse treated with hrANXA1. Magnification = ×20; scale bar = 100 µm. (**E**) Mice treated with hrANXA1 (*n = *8) displayed a significant decrease in tau phosphorylation compared with the vehicle group (*n = *6), in the motor cortex of Tau-P301L mice. Graphs represent density quantification of: AT8 upper band, AT8 lower, AT8 both bands, and representative western blot. Data were normalized to β-actin, and are plotted as mean ± SEM. Statistical analysis was conducted with a one-tailed Student’s independent *t*-test, * *P < *0.05, ****P <* *0.001. (**F**) Western blot results for total tau showing that treatment with hrANXA1 (*n = *8) caused no change in overall tau levels, compared with the vehicle group. Data were normalized to β-actin, and are plotted as mean ± SEM. Statistical analysis was conducted with a one-tailed Student’s independent *t*-test.

In contrast with 5xFAD mice, brain Evans blue dye content measured in the frontal cortex and cerebellum of 7–8-month-old wild-type and Tau-P301L mice after acute intravenous treatment with hrANXA1 or vehicle did not reveal major changes in blood–brain barrier leakage in Tau transgenic mice (Fig. 7A). Similar results were observed by fibrinogen staining (Fig. 7B). Furthermore, confocal images of sections of vehicle-treated Tau-P301L mice fluorescently stained for endogenous IgG showed IgG localized within the blood vessels (white arrows), indicating no extravasation of IgG into the brain parenchyma of Tau-P301L mice (Fig. 7C). Similarly, endogenous IgG was localized within blood vessels (white arrows) of hrANXA1-treated mice, representing no differences between the two groups (Fig. 7C).

To confirm whether hrANXA1 was able to affect vascular integrity in Tau-P301L mice, we carried out FASTClear staining in brain sections of Tau-P301L mice treated with hrANXA1 or vehicle. Double staining with tomato lectin and occludin showed a disruption of tight junctions most apparent in the Tau-P301L mouse treated with vehicle as shown by the sparse, punctate occludin stain in Fig. 7D. By contrast, the occludin signal was much stronger and more consistently co-localized with the lectin staining in the hrANXA1 treated mouse, suggesting that ANXA1 can restore tight junction expression and localization in this model (Fig. 7D).

Following this, we assessed the effect of acute treatment with 0.67 μg/kg hrANXA1 on the expression of phosphorylated (p)-tau and total tau in cortical homogenates of Tau-P301L mice. Interestingly, we detected a reduction in the levels of two isoforms of p-tau in the cortex of animals treated with hrANXA1 with the AT8 (Ser202 and Thr205) antibody (Fig. 7E), without changes in total tau levels (Fig. 7F).

The long-term effects of ANXA1 in a tau model were then analysed in Tau-P301S mice. The results obtained by the fear conditioning paradigm followed the same pattern as for the 5xFAD mice, showing that subchronic treatment with hrANXA1 (1 µg/day intraperitoneally for 7 days) improved hippocampal- and amygdala-dependent memory in transgenic but not in wild-type mice and resulted in an increase in synaptophysin expression (Supplementary Fig. 6).

In conclusion, hrANXA1 treatment appears to have beneficial effects in transgenic models of tau pathology by reducing tau phosphorylation, reversing neurovascular disruption and increasing synaptic markers.

## Discussion

Alterations in blood–brain barrier permeability have been reported in patients with Alzheimer’s disease^5^^,^^38^ and also in animal models of amyloidosis.^39–41^ The adverse effects include infiltration of pro-inflammatory molecules and cells, while recently blood–brain barrier dysfunction was described in Alzheimer’s disease-associated epilepsy.^42^ Here, we investigated blood–brain barrier breakdown and demonstrate that it can be reversed by hrANXA1 administration, resulting in reduced amyloid-β pathology and improved memory in young 5xFAD mice.

Other groups have also investigated blood–brain barrier alterations in 5xFAD mice,^40^ showing differences in blood–brain barrier permeability at age 6–7 months, while our results define blood–brain barrier breakdown as an event earlier in disease progression. One other study described the progressive age-dependent increase in FITC-albumin leakage into the brain of 5xFAD mice, beginning already at the age of 4 months.^39^ Our data establish blood–brain barrier leakiness in young 5xFAD mice, with extravasation of molecules like albumin-bound Evans blue and fibrinogen, which is not detected in older mice. These results are in line with a recent publication showing that the amount of plasma proteins entering the brain actually decreases with age.^43^

The blood–brain barrier permeability in young 5xFAD mice is not caused by CAA, because we and others observed no vascular amyloid-β deposition at a young age in this model.^39^^,^^44^ Alterations in morphology and architecture of the cerebral vasculature were described in ageing APP23 mice, in particular ‘holes’ in the cortical vasculature network associated with amyloid-β plaques^45^ and reductions in the size of microvasculature in Tg4510 mice expressing P301L mutant tau.^4^ On the other hand, amyloid-β aggregates in or at the vessel walls are not a prerequisite for vascular dysfunction (reviewed in Klohs *et al*.^44^) Most likely, the vascular abnormalities in young 5xFAD mice are caused by inflammatory responses associated with amyloid-β oligomerization.^46^ Furthermore, inflammation in Alzheimer’s disease causes increased blood–brain barrier permeability^47^^,^^48^ while even peripheral systemic inflammation was linked to blood–brain barrier dysfunction.^49^^,^^50^

Our results demonstrate the striking disruption of the endothelium in brain blood vessels in young 5xFAD mice, already at 12 weeks of age. Particularly, the observed loss of CD31 (PECAM1) from brain vascular endothelial cells will affect adherens junctions.^51^ Interestingly, endothelial alterations in gene expression profile in neurological pathologies also affects their functionality in transcytosis and transport.^52^

Here, we demonstrate that defects in endothelial cells were reversed by treatment with hrANXA1, which functionally translated into reduced blood–brain barrier leakage. Furthermore, Evans blue extravasation was reversed by hrANXA1 in young 5xFAD mice. Our data extend the report on ANXA1 knockout mice that suffer increased blood–brain barrier permeability, which was also reversed by administration of hrANXA1.^22^ Thereby, hrANXA1 activates the FPR2 receptor to inhibit RhoA signalling, leading to redistribution of tight junction components.^22^ We observed significant changes in the distribution of the tight junction marker occludin in young 5xFAD mice treated with hrANXA1 and Tau-P301L mice treated with hrANXA1. Our *in vivo* data are in line with *in vitro* rescue of amyloid-β-induced blood–brain barrier permeability by hrANXA1 in a brain endothelial cell line, through inhibition of the RhoA/ROCK signalling pathway.^41^

In addition to alterations in tight-junction markers, we detected abnormalities in the distribution of AQP4 in young 5xFAD mice. The loss of perivascular AQP4 has been associated with increased amyloid-β burden in Alzheimer’s disease brain, following impaired clearance.^53^ Furthermore, amyloid-β reduces AQP4 expression in astrocytes *in vitro.*^54^ Others have reported loss of vascular AQP4 in aged TgSwDI mice, another model of Alzheimer’s disease.^55^ The disruption in AQP4 localization appears not to be because of its altered expression levels. The compartmentalization of AQP4 at astrocytic endfeet is complexly regulated by secreted factors, by extracellular matrix components and by the actin cytoskeleton.^56^ Interestingly, ANXA1 enhances actin polymerization *in vitro*,^57^ in addition to inhibition of phosphorylation of p38.^58^

Conversely, alterations in the perivascular distribution of AQP4 could affect amyloid-β clearance via the glymphatic system, although this pathway and the role of AQP4 remain a matter of debate.^59^^,^^60^

Notably, the observed correlations of blood–brain barrier permeability with amyloid-β load in the hippocampus of young 5xFAD mice suggests a close link. On the other hand, ANXA1 could act by increasing the expression of peptidases such as IDE and neprilysin, and by boosting microglial phagocytosis.^24^ Moreover, as recombinant ANXA1 crosses the blood–brain barrier (Supplementary Figs 7 and 8), its neuroprotective effects in young 5xFAD mice could be mediated at the level of microglia, astrocytes, or even neurons. ANXA1 is associated with anti-inflammatory protective actions against toxic mediators such as reactive oxygen species and pro-inflammatory cytokines that exacerbate neuroinflammation in Alzheimer’s disease and other neurodegenerative diseases.^61^^,^^62^ We have previously reported that ANXA1 reduced pro-inflammatory cytokines and induced anti-inflammatory mediators *in vitro* in cells stimulated with synthetic amyloid-β,^24^ and in the present study we observed some limited effects on neuroinflammatory markers by a single dose in young 5xFAD mice.

Furthermore, our results show that acute administration of hrANXA1 in a transgenic mouse model of tauopathy can reduce build-up of p-tau in the cortex. This could potentially be due to a regulatory effect of ANXA1 on tau kinases, although we did not detect changes in the expression of glycogen synthase kinase-3 (data not shown). However, disrupted calcium homeostasis in Alzheimer’s disease leads to an aberrant activity of other tau kinases, such as CDK5, causing tau phosphorylation. As ANXA1 has a regulatory effect on calcium release, it may thus reduce CDK5 activity indirectly.^63^

Interestingly, subchronic administration of hrANXA1 increased synaptic density and rescued memory deficits in young 5xFAD and Tau-P301S mice. ANXA1 was suggested to be neuroprotective by attenuating microglial activation.^17^ We hypothesize that its anti-inflammatory properties, potentially by reducing T-cell infiltration, mediate its positive effects on synapses and memory. Alternatively, improved synaptic plasticity can be secondary to reduced levels of amyloid-β oligomers, known to impair synaptic plasticity.^64^ Moreover, the restored vascular integrity with improved blood supply to the brain will evidently improve neuronal functions, as will VEGF expression or other means.^65^

Finally, ANXA1 regulates glucose metabolism and is proposed to be involved in diabetes, as ANXA1 knockout mice suffer a diabetic phenotype.^66^^,^^67^ Interestingly, the baseline differences in 5xFAD mice detected by CEST imaging can be ascribed to accumulated glucose and its defective metabolism,^68^ which were reversed by hrANXA1 treatment. Nevertheless, several other endogenous exchangeable entities contribute to the CEST signal, including glycosaminoglycans, glycogen, myo-inositol, and lactate.^69–73^

In conclusion, we note that hrANXA1 presents several advantages compared to therapeutics in development, in particular in promoting neurovascular integrity. Endothelial dysfunction, with its associated white matter abnormalities, is of utmost importance in the pathology of dementia. Drugs that stabilize endothelial dysfunction in small vessel disease reversed the endothelial and oligodendroglial pathology, presenting as a new therapeutic option.^74^ The obvious importance of maintaining or repairing blood–brain barrier integrity in Alzheimer’s disease is a most valuable target, whereby hrANXA1 could be of great potential.

## Supplementary Material

awab050_Supplementary_Data

## Abbreviations

CEST: chemical exchange saturation transfer; hrANXA1 = human recombinant ANXA1
