# Supplementary material for: Annexin A1 restores cerebrovascular integrity concomitant with reduced amyloid-β and tau pathology
Source: Brain. 2021 Jun 21;144(5):1526–41. doi: 10.1093/brain/awab050 (PMC8262982; doi:10.1093/brain/awab050)
Supplement: awab050_Supplementary_Data [file awab050_supplementary_data.zip › awab050-suppl_data/awab050_Supplementary_material.pdf]

Supplementary material

Materials and Antibodies

Antibodies used were 6E10 against anti-Aβ MOAB-2 (6C3) (Millipore), Aβ1-16 (Covance), anti-BACE1 antibody (Cell Signalling), anti-neprilysin antibody (Santa Cruz), anti-insulin degrading enzyme (IDE) antibody (Abcam), anti-LRP1 antibody (kind gift from Prof. Claus Pietrzik, Johannes-Gutenberg-University, Mainz, Germany), anti-AQP4 antibody (Santa Cruz), anti-laminin antibody (Sigma), anti-PECAM1 antibody (BD Biosciences), anti-occludin antibody (Invitrogen), anti-Iba1 antibody (Wako), anti-GFAP rabbit antibody (Abcam), anti-GFAP rat antibody (Invitrogen), anti-synaptophysin antibody (Millipore), anti-ApoE antibody (Santa Cruz), anti-CD3 antibody (DAKO), anti-p-tau AT8 (Thermofisher Scientific), anti-total tau (DAKO), anti-fibrinogen (Dako), anti-GAPDH antibody (Santa Cruz), and anti-β-actin antibody (Abcam). Reagents were purchased from Thermo Fisher Scientific or from Sigma-Merck, UK, unless stated otherwise.

Western blotting

Brain tissue was homogenized and protein extracted with RIPA (1 % Triton X-100, 1 % sodium deoxycholate, 0.1 % SDS, 150 mM NaCl, and 50 mM Tris-HCl, pH 7.2) supplemented with cOmplete protease inhibitor (Roche) and phosphatase inhibitor (Roche). Equal amounts of protein were loaded in 10% SDS-PAGE gels and transferred to nitrocellulose (GE) or PVDF (Millipore) membranes before being blotted with primary antibodies and detected with HRP-conjugated secondary antibodies in 5% non-fat dried milk or 5% BSA in TBS with 0.5% Tween (TBST). Blots were developed with ECL reagents (GE Amersham), followed by visualisation using ECL Hyperfilm with an automated developer (Konika), or a GeneGnome Chemiluminescent imaging system (Syngene). To re-probe membranes using a different antibody, blots were stripped with ReBlot Plus Strong Antibody Stripping Solution (Millipore).

Digital Western blot images were quantified using the Gel Analyzer tool in ImageJ and normalised to β-actin or GAPDH, or full-length APP for APP cleavage products to control for protein loading.

ELISA

Enzyme-linked immunosorbent assays (ELISA) were carried out according to the manufacturers' instructions using kits for murine TNFα, IL-1β, IL-10, and IFNγ (Peprotech). Aβ subtypes were determined by ELISA using high sensitivity human Aβ ELISA kits (Millipore). A total protein concentration of 10 mg/ml of brain homogenates was loaded for Aβ ELISA and 50 - 500 mg/ml for cytokine ELISA. Serum was diluted 1:10 for analysis. Values obtained were normalised to overall protein concentration for tissue homogenates and expressed as pg/mg protein or expressed as pg/ml for serum investigations.

Fear conditioning

Mice were accustomed to being handled through daily handling in the 7 days before the start of chronic hrANXA1 treatment. Fear conditioning memory testing was carried after chronic treatment with hrANXA1 or vehicle in 3-month-old 5xFAD and 5-6 month-old tau P301S mice and WT littermates (Fig. S5 and S6). Trace fear conditioning was carried out as previously described (Katsouri *et al.*, 2013; Vizcaychipi, Lloyd, *et al.*, 2011; Vizcaychipi, Xu, *et al.*, 2011), with a conditioned stimulus (CS) paired with a delayed unconditioned stimulus (US) thus allowing an estimate of both amygdala and hippocampal-dependent memory (Rogers *et al.*, 2006). Briefly, during the training phase, after an acclimatisation time of 2 min, mice were exposed to a 40 s conditioned stimulus (CS, 2000 Hz, 76 dB tone) followed by a 20 s trace interval and a 2 s unconditioned stimulus (US, 0.5 mA foot shock) in the fear conditioning chamber (Ugo Basile) (Fig. S5A). After a variable delay of 30-45 s, this was repeated such that a total of 4 CS-US pairings were presented to each animal. The next morning, mice were

exposed to the same context (white walls, standard cleaning spray, fan speed 80%) for 5 minutes to test context-dependent memory (Fig. S5B, S6). In the afternoon, mice were placed in a novel context (striped walls, lemon cleaning spray, fan off) and the response to the CS assessed to determine conditional memory during the CS (amygdala-dependent and hippocampal-independent) and the hippocampal-dependent response during the 30 s after the end of CS presentation (trace interval) (Fig. S5D, S6). During behavioural tests, videos were acquired and tracked with EthoVision XT 10.1 software to determine the animals' proportion of inactivity (freezing) in each time segment, with a threshold for freezing set at 0.15% activity, exclude instances shorter than 0.1 s. Freezing was defined as the absence of any visible movement, except that required for respiration. The following time segments were analysed: training - during CS1-4 and trace interval 1-4; contextual - whole interval; conditional – acclimatization time, during CS, and during 30 s after CS. Percentage freezing was normalized to baseline freezing.

In vivo and ex-vivo imaging and analysis

Animals were scanned 24 h after acute hrANXA1 treatment and were fasted for 16 h prior to scanning then anaesthetised with isofluorane. The tail vein was cannulated with a catheter and the dead volume minimised to 20µL. A T-connector (30 µL volume) with three lines was connected to a 1 mmol/ml Gd-DTPA (gadobutrol (Gadovist®)) solution, a 0.5 g/mL D-glucose solution and the tail vein. Animals were placed in a 9.4 T Bruker BioSpec scanner equipped with a 4-channel phase array receiver coil. After localization scans, two GlucoCEST baseline Z spectra were acquired using a single slice RARE readout (TR/TE=4s/6ms, RARE factor=23, 1mm thickness, centric encoding, ~2.5min acquisition time) and one hard CEST prepulse (3s duration, B1=1.6 µT, ±3.2 ppm, 17 offsets). A total volume of 200µl D-glucose bolus was injected at 0.15 ml/min using a syringe pump placed inside the MRI room and three subsequent glucoCEST arrays were acquired to track the bolus passage. Water saturation shift referencing

(WASSR) (Kim *et al.*, 2009)was also acquired for B0 inhomogeneity correction. DCE images were acquired 30min post-glucose injection. A total volume of 100 μl Gd-DTPA was injected at 0.6 ml/min controlled by the syringe pump. Multi-slice T1-weighted images for Gd-DTPA-dynamic contrast enhancement (DCE) were captured for 3 minutes preceding and 10 minutes following Gd-DTPA injection with a multi-slice fast low-angle shot (FLASH) sequence (TE/TR/flip angle/temporal resolution=1.21 ms/26 ms/70º/1.4 s). Susceptibility weighted imaging (SWI) offers a unique contrast (Haacke et al., 2004), different from the classical T1, T2 and T2*, and was used in this study to visualize the blood vessel architecture of the mouse brain. SWI images were acquired after DCE with a velocity compensated gradient echo sequence having the following parameters: TR/TE = 550/12 ms, FOV = (16 × 16) mm^2, 400 μm slice thickness, matrix size = (266 × 266) resulting in an in plane spatial resolution of (60 × 60) μm^2, and total scan time of 9 min 45 s. To reduce susceptibility artefacts caused by B_0 field inhomogeneities, fieldmap based shimming was performed before the acquisition (up to 4th order) in a predefined region of interest covering the entire mouse brain.

Relative contrast enhancement (RCE) maps were generated with DCE@urLAB software (Ortuño et al., 2013) using a manually drawn whole brain ROI. RCE was calculated by dividing the maximum value by the minimum value and multiplying by 100%. The average of two consecutive slices was taken per mouse.

GlucoCEST maps were generated using Matlab (The Mathworks), asymmetry Z-spectra were corrected for B0 inhomogeneity using the WASSR spectra, and integral 0.8-2.2ppm maps were calculated. ROIs were drawn to match the DCE ROIs.

For SWI, histograms of signal distribution were initially computed ranging from pixel values of 0 to 100 a.u. with 60 binning intervals. Given that the most heterogeneous distribution was observed at low intensity pixel values, a denser distribution (150 bins) was subsequently

performed, and a threshold was set to an interval of 0 to 5 a.u. pixel value. Pixel count analysis was done for the three groups (WT, 5xFAD untreated and 5xFAD treated mice) to determine whether there is a difference between the three groups. Higher number of pixels of low intensity values is an indication of SWI contrast darkening, reflective of micro-haemorrhage.

For visualization of FL-hrANXA1, brains were imaged *ex vivo* immediately following pentobarbital overdose at 15min post-injection and transcardial perfusion with PBS. Brain epifluorescent images were acquired simultaneously with a Caliper IVIS Lumina XR imaging system (Perkin Elmer) and calculated as radiant efficiency (photons/sec/cm2/sr/μW/cm2) displayed in pseudocolour using the Living Image 4.3 software.

Immunostaining imaging and analysis

Tiled images of immunostaining of whole sections were acquired using Eclipse 80i camera (Nikon) and ImagePro software (Media Cybernetics) or Eclipse E800 microscope (Nikon), digital camera (Qimaging), and Surveyor software (Objective Imaging). For CD3 staining, images were obtained using a scanner Leica Aperio AT2. Images of immunofluorescence staining were acquired using an LSM 780-inverted confocal laser scanning microscope (Carl Zeiss), and ZEN software (Carl Zeiss).

All image analysis was carried out using the Fiji distribution of ImageJ (Schindelin et al., 2012), investigators were blinded to treatment group. Percentage area staining was determined by manually setting a threshold, with regions of interest determined based on the P56 sagittal Allen Mouse Brain Atlas (Lein et al., 2007). Co-localisation was analysed in Z-stacks using the Co-localisation Threshold plugin. PECAM1 staining was quantified by measuring signal intensity in manually drawn regions of interest of vascular structures in maximum Z-projections. Plaque-associated microglia and astrocytes were analysed by placing a circular 50 mm Ø ROI centred on each ThioS-positive plaque on maximum Z-projection. Thresholds were

manually adjusted for each channel and %area as well as intensity within each ROI were determined for the GFAP and Iba1 staining channels. Synaptophysin staining intensity was analysed by manual adjustment of threshold and measurement of %area as well as intensity on maximum Z-projections. For CD3+ cell quantification, subiculum area was measured and for each section and the number of positive cells in the subiculum was manually quantified using the analyse cell counter plugin.

Experiments of in vitro endocytosis

Primary murine brain endothelial cells isolated as described previously (Cristante *et al.*, 2013) were transferred from flasks to Lab-Tek chamber slides (Sigma-Aldrich). Cells were directly incubated with FITC-ANXA1 for 1, 5 and 15 minutes. Negative controls were prepared by omitting the FITC-ANXA1 incubation step. Subsequently, the cells were transferred at 4°C, washed with PBS, fixed with PFA at 2% in PBS for 10 minutes and stored in PBS at 4°C overnight. The following day the cells were permeabilized with 0.02% Saponin in PBS and stained with Phalloidin 568 (Invitrogen), diluted 1:40 in PBS, at RT for 30 minutes. After washing with PBS, the cells were counterstained with TOPRO3 (Invitrogen) diluted 1:5k in PBS for 10 minutes and coverslipped with Vectashield mounting medium (Vector Laboratories). The cells were examined under Leica TCS SP5 confocal laser scanning microscope (Leica Microsystems) using a sequential scan procedure. Confocal images were taken at 0.35 μm intervals through the x-, y- and z- axes of the section, with 40x and 63x oil lenses.

References

Haacke EM, Xu Y, Cheng Y-CN, Reichenbach JR. Susceptibility weighted imaging (SWI). Magn Reson Med 2004; 52: 612–8.

Katsouri L, Vizcaychipi MP, McArthur S, Harrison I, Suárez-Calvet M, Lleó A, et al. Prazosin, an α(1)-adrenoceptor antagonist, prevents memory deterioration in the APP23 transgenic mouse model of Alzheimer's disease. Neurobiol Aging 2013; 34: 1105–15.

Kim M, Gillen J, Landman BA, Zhou J, van Zijl PCM. Water saturation shift referencing (WASSR) for chemical exchange saturation transfer (CEST) experiments. Magn Reson Med 2009; 61: 1441–50. AQ8

Lein ES, Hawrylycz MJ, Ao N, Ayres M, Bensinger A, Bernard A, et al. Genome-wide atlas of gene expression in the adult mouse brain. Nature 2007; 445: 168–76.

Ortuño JE, Ledesma-Carbayo MJ, Simões RV, Candiota AP, Arús C, Santos A. DCE@urLAB: a dynamic contrast-enhanced MRI pharmacokinetic analysis tool for preclinical data. BMC Bioinformatics 2013; 14: 316.

Schindelin J, Arganda-Carreras I, Frise E, Kaynig V, Longair M, Pietzsch T, et al. Fiji: an open-source platform for biological-image analysis. Nat Methods 2012; 9: 676–82.

Vizcaychipi MP, Lloyd DG, Wan Y, Palazzo MG, Maze M, Ma D. Xenon pretreatment may prevent early memory decline after isoflurane anesthesia and surgery in mice. PLoS One 2011; 6: e26394.

Vizcaychipi MP, Xu L, Barreto GE, Ma D, Maze M, Giffard RG. Heat shock protein 72 overexpression prevents early postoperative memory decline after orthopedic surgery under general anesthesia in mice. Anesthesiology 2011; 114: 891–900.

Supplementary figures

Figure S1

A) Representative maximum Z projection images of immunofluorescent staining for AQP4 (green) and lectin (red) in the cortex and hippocampus in 1 mm coronal brain sections of WT and 5xFAD mice treated with hrANXA1 or vehicle after tissue clearing.
B) Representative Western blots and quantitative analysis of AQP4 expression in the motor cortex of WT and 5xFAD mice treated with hrANXA1 or vehicle detected by AQP4(H-80) antibody, normalised to β-actin (n=6/group). Columns represent mean±SEM.
C) Representative images of of immunofluorescent staining for fibrinogen (green) and lectin (red) in the cortex of WT and 5xFAD mice treated with hrANXA1 or vehicle.

Figure S2

hrANXA1 does not affect measures of Aβ generation or degradation in the hippocampus of 5xFAD mice

A) Representative Western blots and quantification of BACE1 and β-CTF expression in the hippocampus of 5xFAD mice treated with hrANXA1 or vehicle, normalised to β-actin and to flAPP respectively (n=6/group).
B) Representative Western blots and quantification of Neprilysin expression in the hippocampus of 5xFAD mice treated with hrANXA1 or vehicle, normalised to β-actin (n=6/group).
C) Representative Western blots and quantification of IDE expression in the hippocampus of 5xFAD mice treated with hrANXA1 or vehicle, normalised to GAPDH (n=6/group).
D) Representative Western blots and quantification of LRP1 expression in the motor cortex and hippocampus of 5xFAD mice treated with hrANXA1 or vehicle, normalised to β-actin (n=5-6/group).
E) Representative Western blots and quantification of ApoE expression in the motor cortex of 5xFAD mice treated with hrANXA1 or vehicle, normalised to β-actin (n=9/group).
   Columns represent mean±SEM.

Figure S3

hrANXA1 treatment does not affect amyloid pathology in 6-month-old 5xFAD mice

A) Representative images and quantification of % area of ThioS staining in 40 µm brain sagittal sections from 6-month-old mice treated with vehicle or hrANXA1 (0.67 µg/kg) (n=4-5 mice/group, mean 4-6 sections analysed/mouse)..
B) Representative images and quantification of % area of 6C3 staining in 40 µm sagittal brain sections from 6-month-old mice treated with vehicle or hrANXA1 (0.67 µg/kg) (n=4-5 mice/group, mean of 4-6 sections analysed/mouse).
C) Representative Western blots and quantitative analysis of BACE1 (n=5-6/group) and β-CTF (n=6-7/group) expression in the cortex of in 6-month-old mice, normalised to β-actin and flAPP, respectively.
D) Representative Western blots and quantitative analysis of neprilysin and IDE expression in the cortex of in 6-month-old mice (n=6-7/group), normalised to β-actin.
E) Quantification of Evans blue dye assay of BBB permeability 24 h after treatment of 6-month-old WT and 5xFAD mice with vehicle or hrANXA1 (0.67 g/kg i.v., normalised to serum dye content and brain tissue weight in the frontal cortex (n=7-10), hippocampus (n=7-10), and cerebellum (n=7-10).
Columns represent mean±SEM.

Figure S4

Acute hrANXA1 treatment does not affect the expression of GFAP and Iba1

A) qPCR analysis of mRNA expression in the frontal cortex of 5xFAD mice treated with hrANXA1 or vehicle. *Tnf* (n=7-11), *Il6* (n=4-6), *Il4* (n=5-6), *Il1b* (n=5-6), *Arg1* (n=7-10), *Tgfb1* (n=4-6), *Nos2* (n=5-6).
B) Representative Western blots and quantitative analysis of Iba1 expression in the motor cortex (n=6/group) and hippocampus (n=6/group) of 5xFAD mice treated with hrANXA1 or vehicle, normalised to GAPDH and β-actin, respectively.
C) Representative Western blots and quantitative analysis of GFAP expression in the motor cortex (n=9/group) and hippocampus (n=5-6/group) of 5xFAD mice treated with hrANXA1 or vehicle, normalised to β-actin.
D) Representative maximum Z-projection images and quantification of percentage area of immunofluorescent staining for GFAP (magenta), Iba1 (red), and ThioS (green) in a 50 μm diameter circular ROI around plaques in the cortex and subiculum of the hippocampus (n=4 mice/group, mean of 2-3 sections analysed/mouse) of 5xFAD mice treated with hrANXA1 or vehicle.
Columns represent mean±SEM.

Figure S5

Schematic illustration of fear conditioning and effects of hrANXA1 treatment on learning.

A) Schematic of fear conditioning training paradigm.
B) Schematic of fear conditioning contextual test paradigm.
C) Legend indicating conditions for fear conditioning training and testing paradigms.
D) Schematic of fear conditioning conditional test paradigm.
E) Freezing during CS presentation in fear conditioning training after treatment, indicative of hippocampal-independent, amygdala-dependent learning (n=5-9/group). Two-way ANOVA with Bonferroni's multiple comparisons post-test. Symbols indicate statistically significant differences when comparing each instance of stimulus presentation (CS2-CS4) to first CS presentation (CS1) within each group (*=WT Veh, +=WT hrANXA, #=5xFAD Veh, $=5xFAD hrANXA1).
F) Freezing during CS-US trace interval in fear conditioning training after treatment, indicative of hippocampal-dependent learning (n=5-9/group). Two-way ANOVA with Bonferroni's multiple comparisons post-test. Symbols indicate statistically significant differences when comparing each trace interval (trace interval 2-trace interval 4) to first trace interval (trace interval 1) within each group (*=WT Veh, +=WT hrANXA1, #=5xFAD Veh, $=5xFAD hrANXA1).

Columns represent Mean±SEM.

CS=conditioned stimulus, US=unconditioned stimulus.

Figure S6

Sub-chronic hrANXA1 treatment improves hippocampal-dependent memory in tau P301S mice

A) Freezing during CS presentation in fear conditioning training after treatment, indicative of hippocampal-independent, amygdala-dependent learning (n=5-6/group). Two-way ANOVA with Bonferroni's multiple comparisons post-test. Symbols indicate statistically significant differences when comparing each instance of stimulus presentation (CS2-CS4) to first CS presentation (CS1) within each group (+=WT hrANXA1, $=TauP301S hrANXA1).

B) Freezing during CS-US trace interval in fear conditioning training after treatment, indicative of hippocampal-dependent learning (n=5-9/group). Two-way ANOVA with Bonferroni's multiple comparisons post-test. Symbols indicate statistically significant differences when comparing each instance of stimulus presentation (CS2-CS4) to first CS presentation (CS1) within each group (+=WT hrANXA1, $=TauP301S hrANXA1).

C) Freezing in context in which CS-US pair training was carried out, indicative of contextual memory (n=5-6/group). A=acclimatisation time before fear condition training, T=contextual testing. Ratio paired one-tailed t-test.

D) Freezing in novel context during acclimatisation time of conditional test (A) and CS presentation (C), n=5-6/group. Ratio paired one-tailed t-test.

E) Freezing in novel context during acclimatisation time of conditional test (A) and trace interval (T), n=5-6/group. Ratio paired one-tailed t-test.

F) Representative Western blots and quantification of synaptophysin expression in cortex and hippocampus homogenates of WTs and tauP301S mice treated with vehicle or ANXA1 (n=4-6 per group).Student's t test.

Columns represent Mean±SEM. *P<0.05; **P<0.01; ***P<0.001

Figure S7

Fluorescently-labelled hrANXA1 enters the brain parenchyma of 5xFAD mice.

A) IVIS epifluorescent imaging of ex-vivo brains perfused at 15min after intravenous injection indicated higher epifluorescence efficiency in mice receiving FL-hrANXA1 compared with vehicle (VEH) injection.

B and C) Sagittal slices revealed fluorescent signal in FL-hrANXA mice that was not present on VEH sections. Highlighted region of intense fluorescence in C is magnified in

D) illustrating that parenchymal FL-hrANXA1 leaks extended over considerable distances and could mostly be traced to vascular rather than ventricular structures. Magnifications showing that parenchymal FL-hrANXA1 was typically associated with medium-sized vessels (approximately 6-15mm) rather than capillaries.Panel showing perivascular clearance of FL-hrANXA along longitudinal hippocampal vessels in a section containing a diffuse parenchymal leak in adjacent hippocampus (circled in G).

Figure S8

Representative images of endocytosis assays. Filaments of actin, contained in primary murine BECs, are stained with Phalloidin TRITC-conjugated dye. Primary BECs are incubated with green-fluorescent ANXA1, then fixed after 1, 5 or 15 minutes, and permeabilized. Images are documented with fluorescence confocal microscopy (A-D).

A) Cells without FITC-ANXA1 incubation.

B) After 1 minute of incubation, green-fluorescent ANXA1 is detectable at cells surface (white arrowheads).

C) After 5 minutes of incubation, FITC-ANXA1 is internalized, as showed by the typical punctate pattern of endocytosed proteins.

D) After 15 minutes, only few green spots are observable inside the cytoplasm of the BECs. Nuclear counterstaining with TO-PRO3.

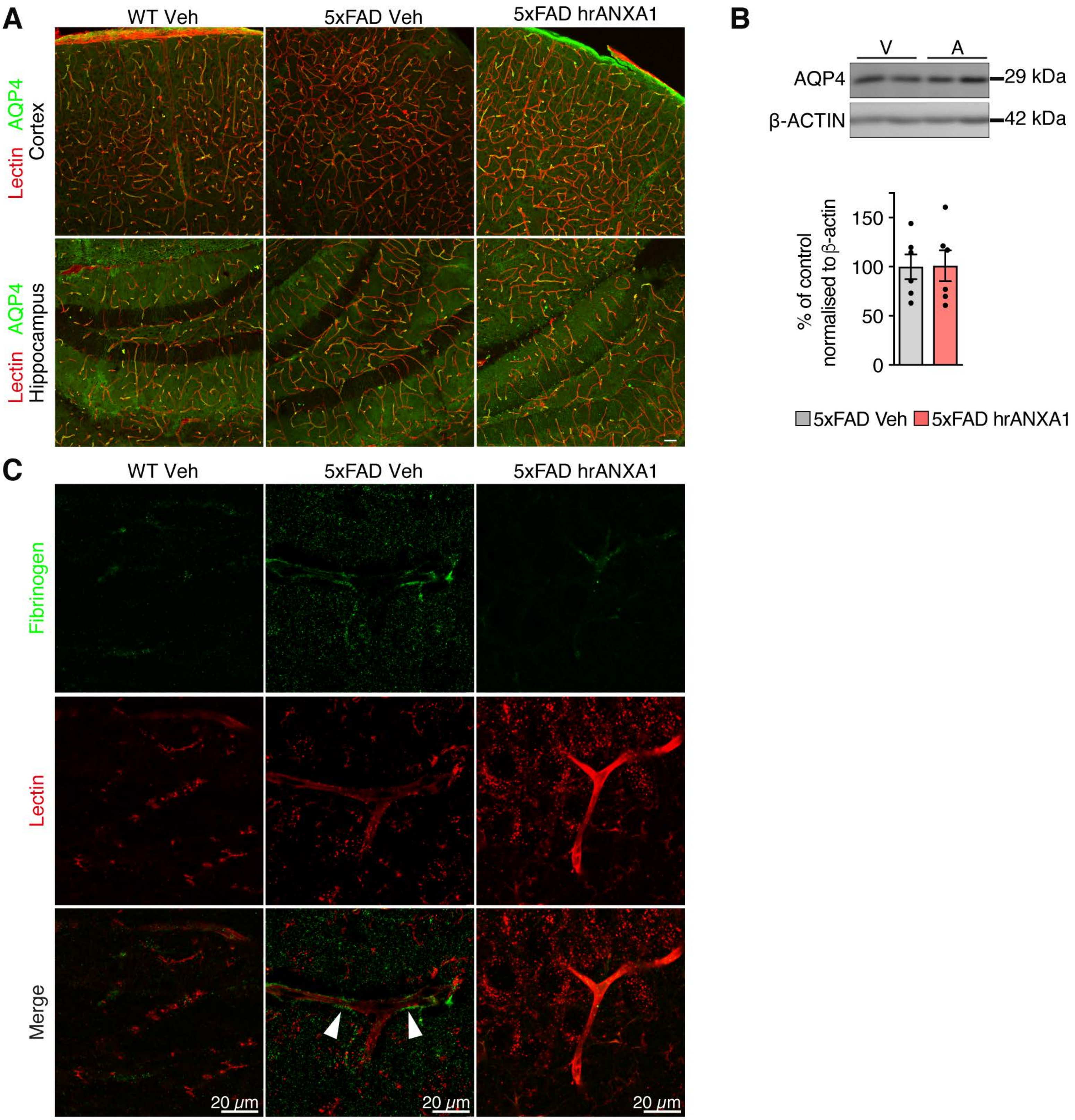

Supplementary Figure 1

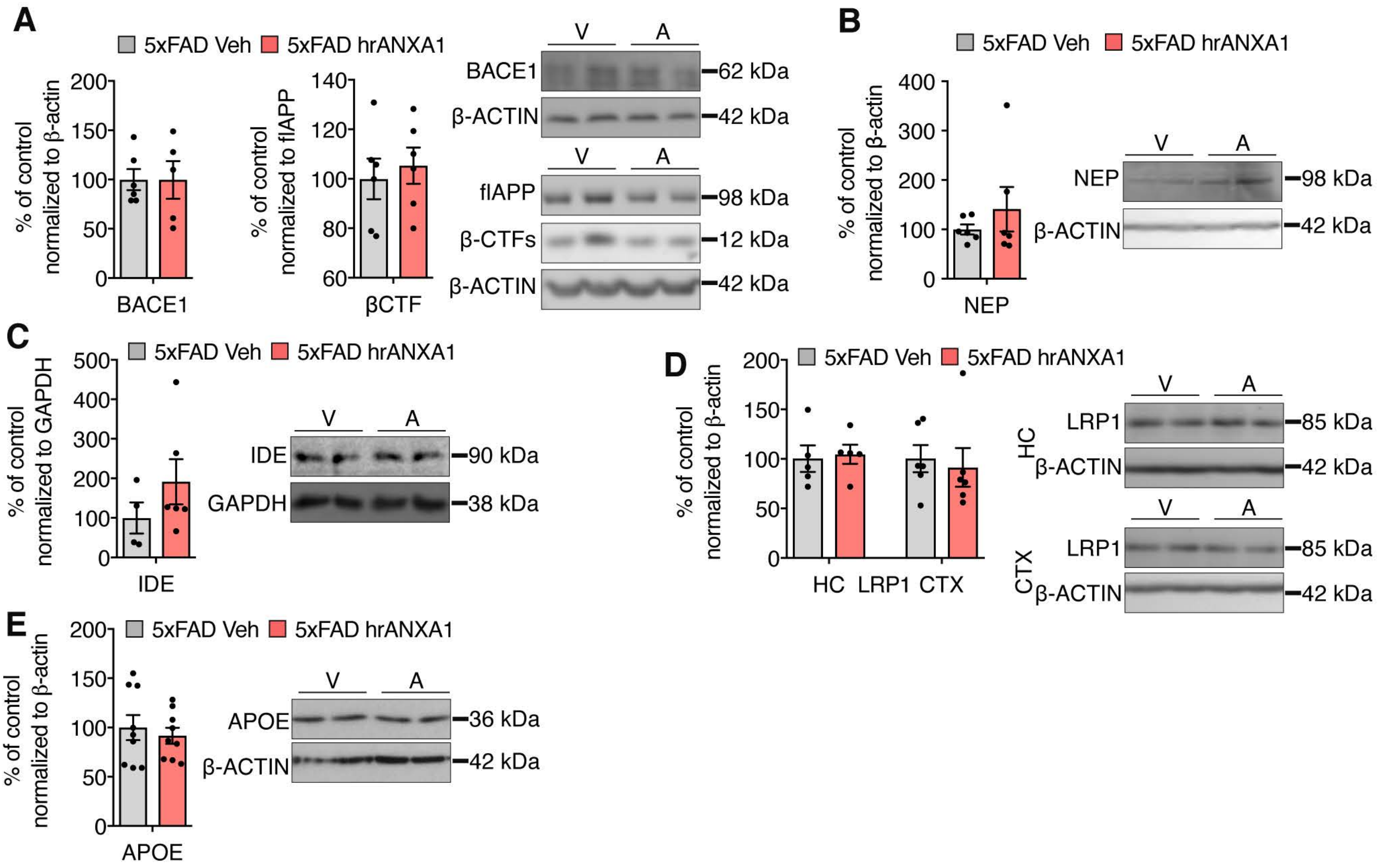

Supplementary Figure 2

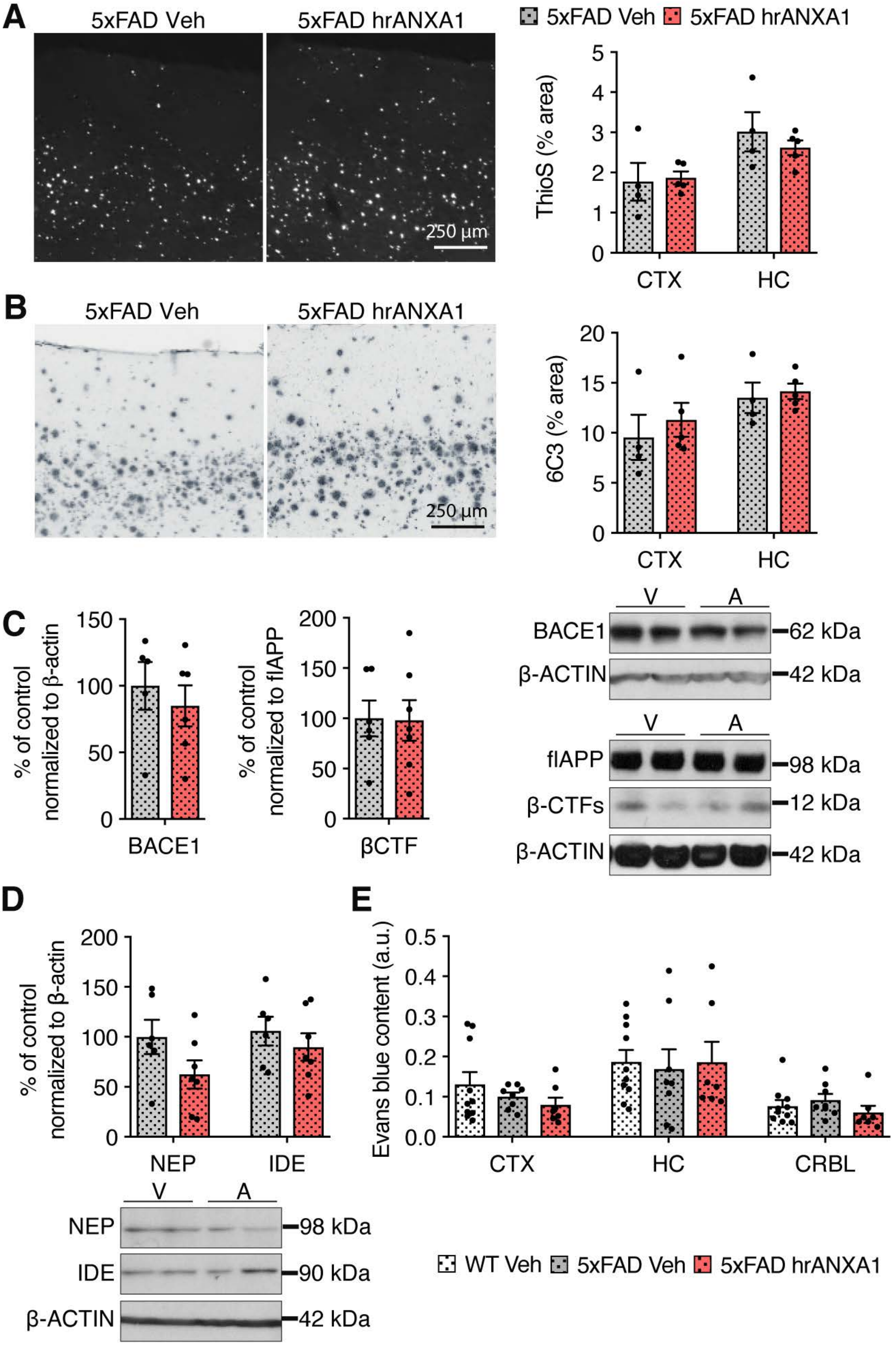

Supplementary Figure 3

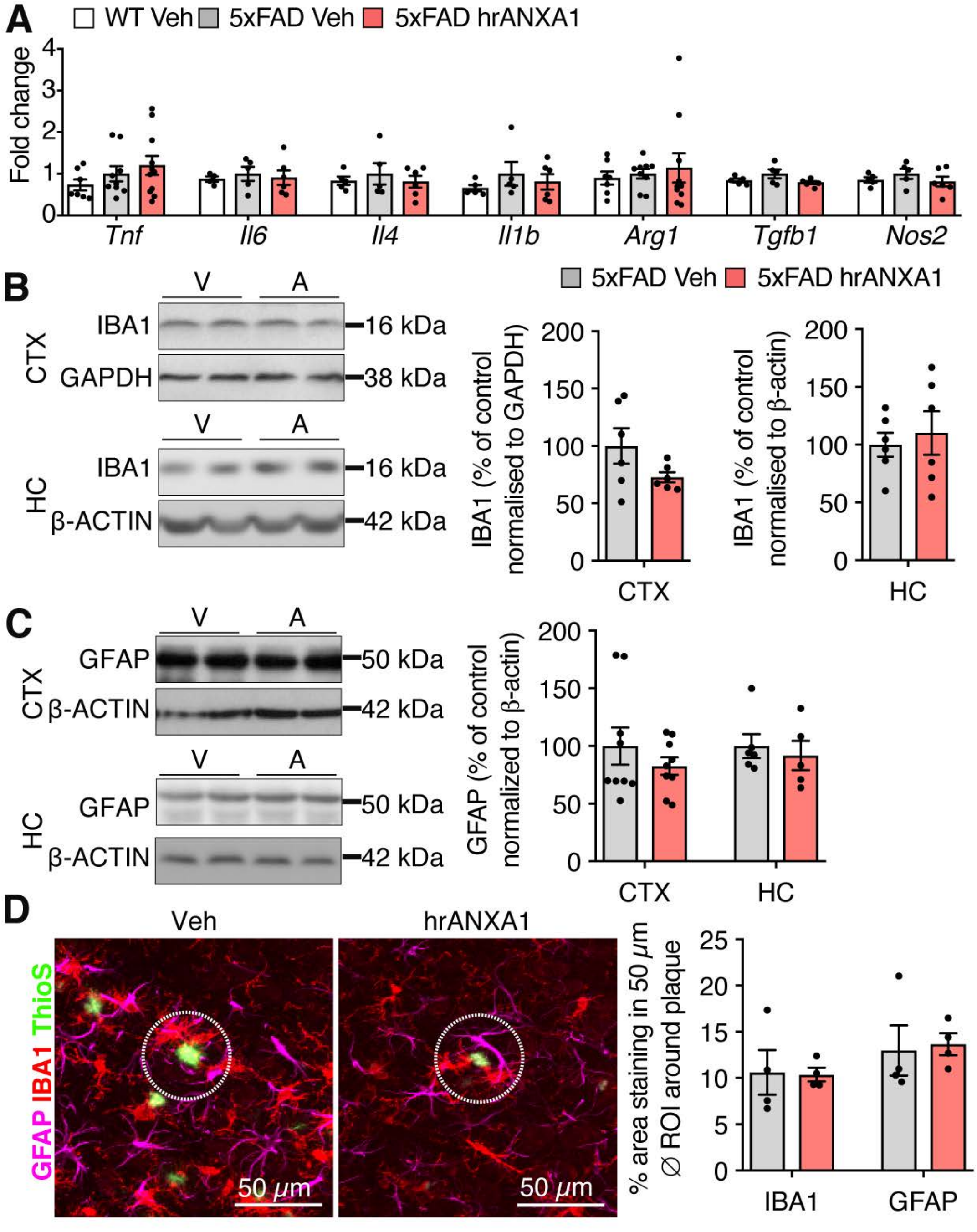

Supplementary Figure 4

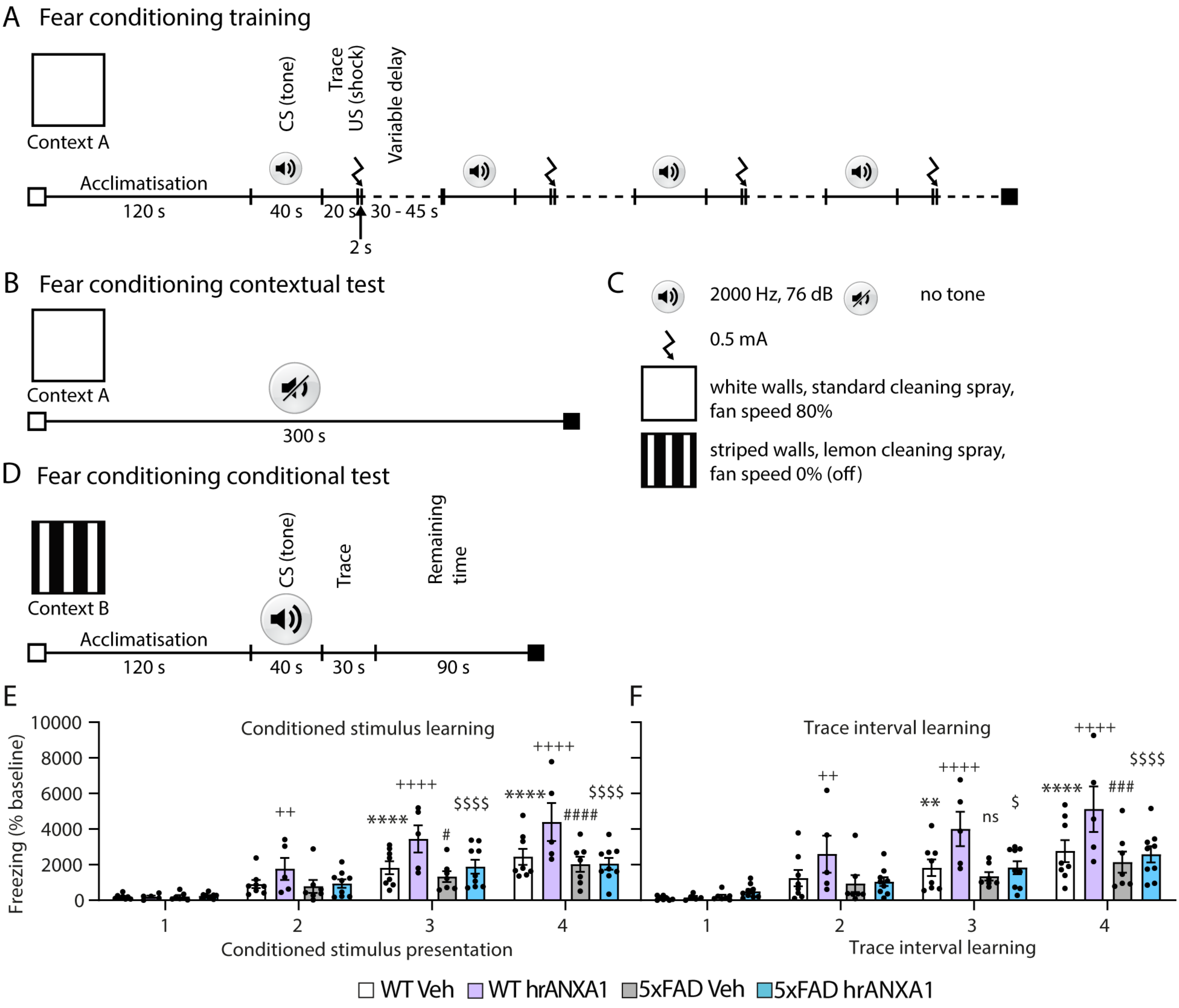

Supplementary Figure 5

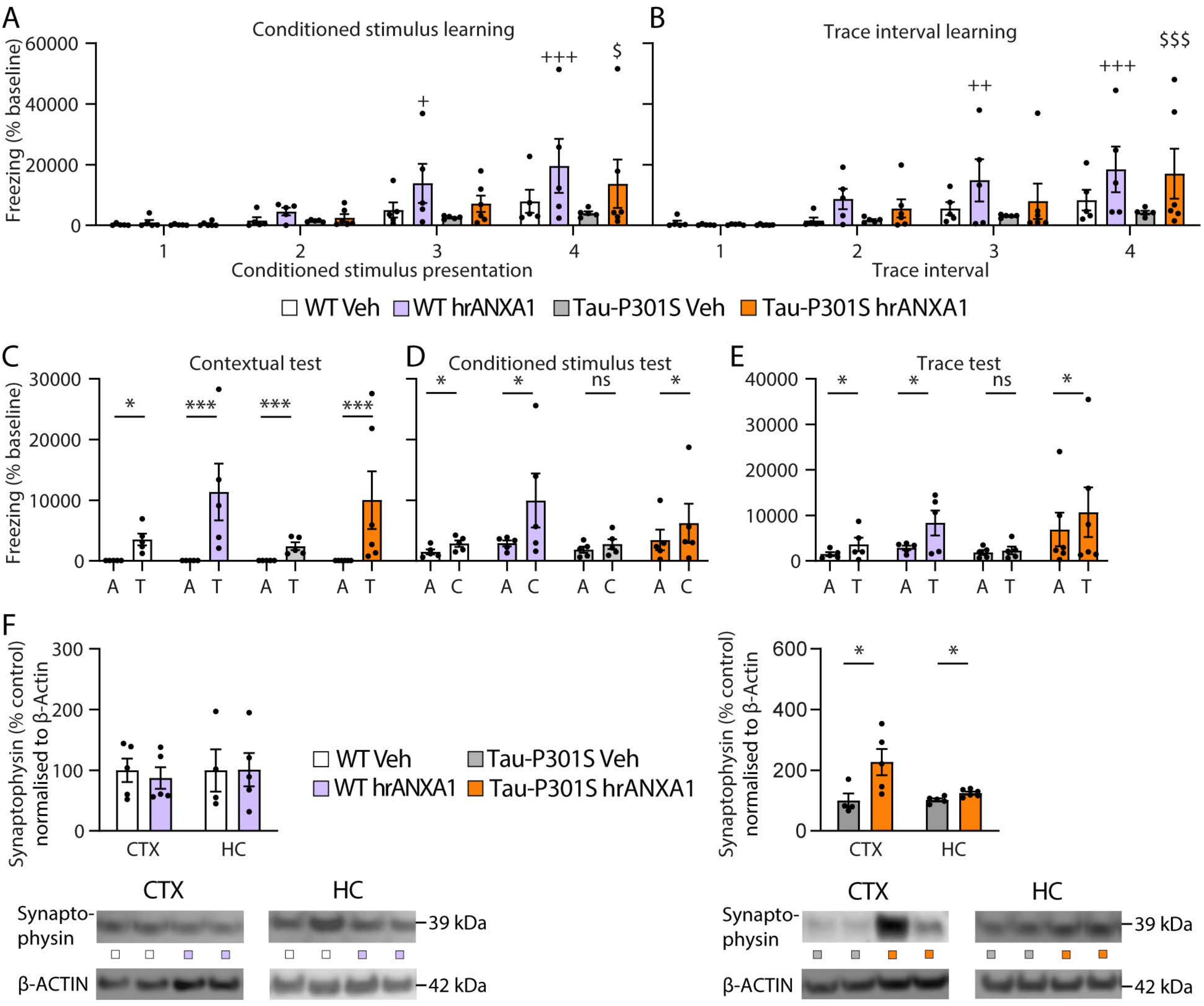

Supplementary Figure 6

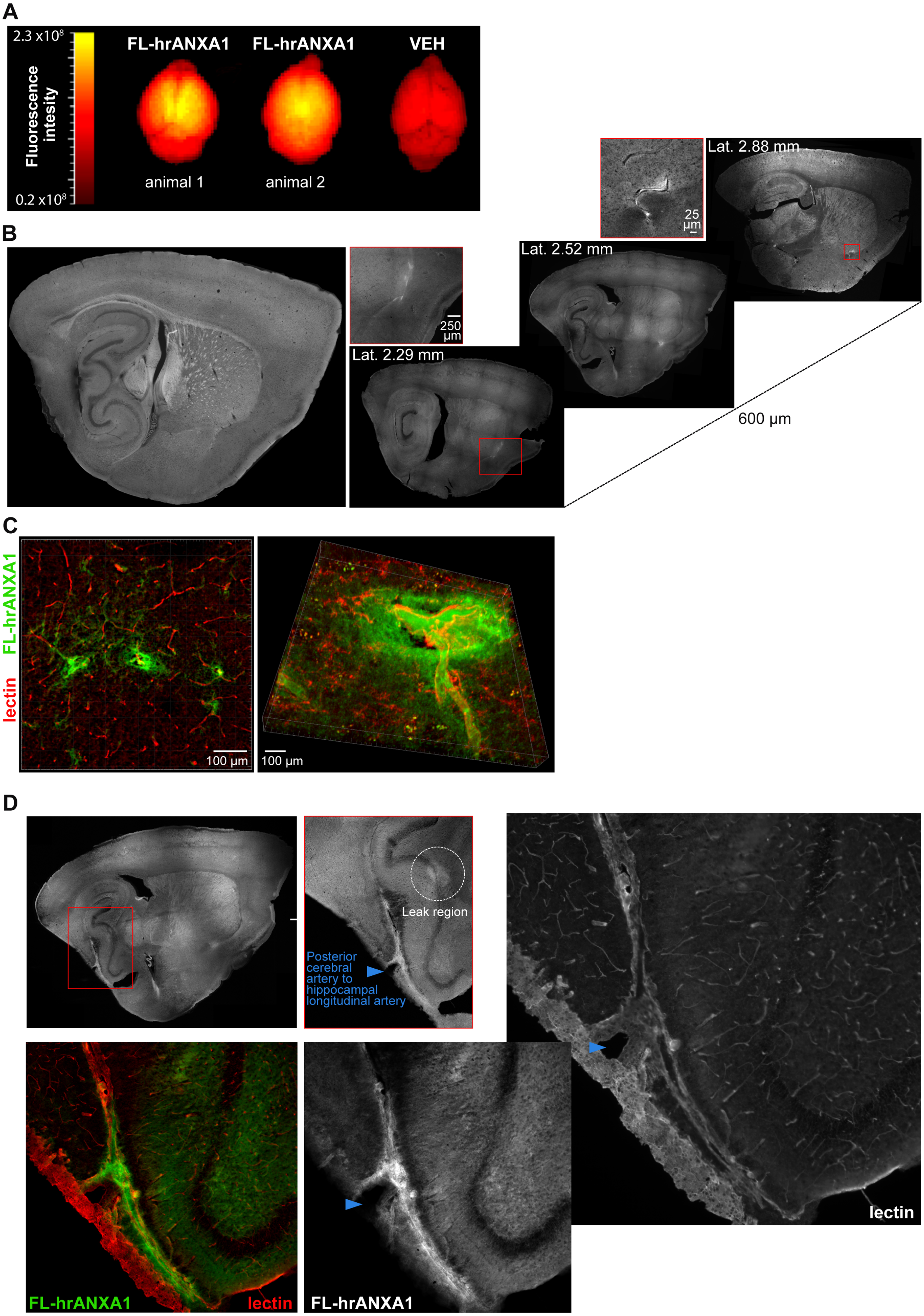

Supplementary Figure 7

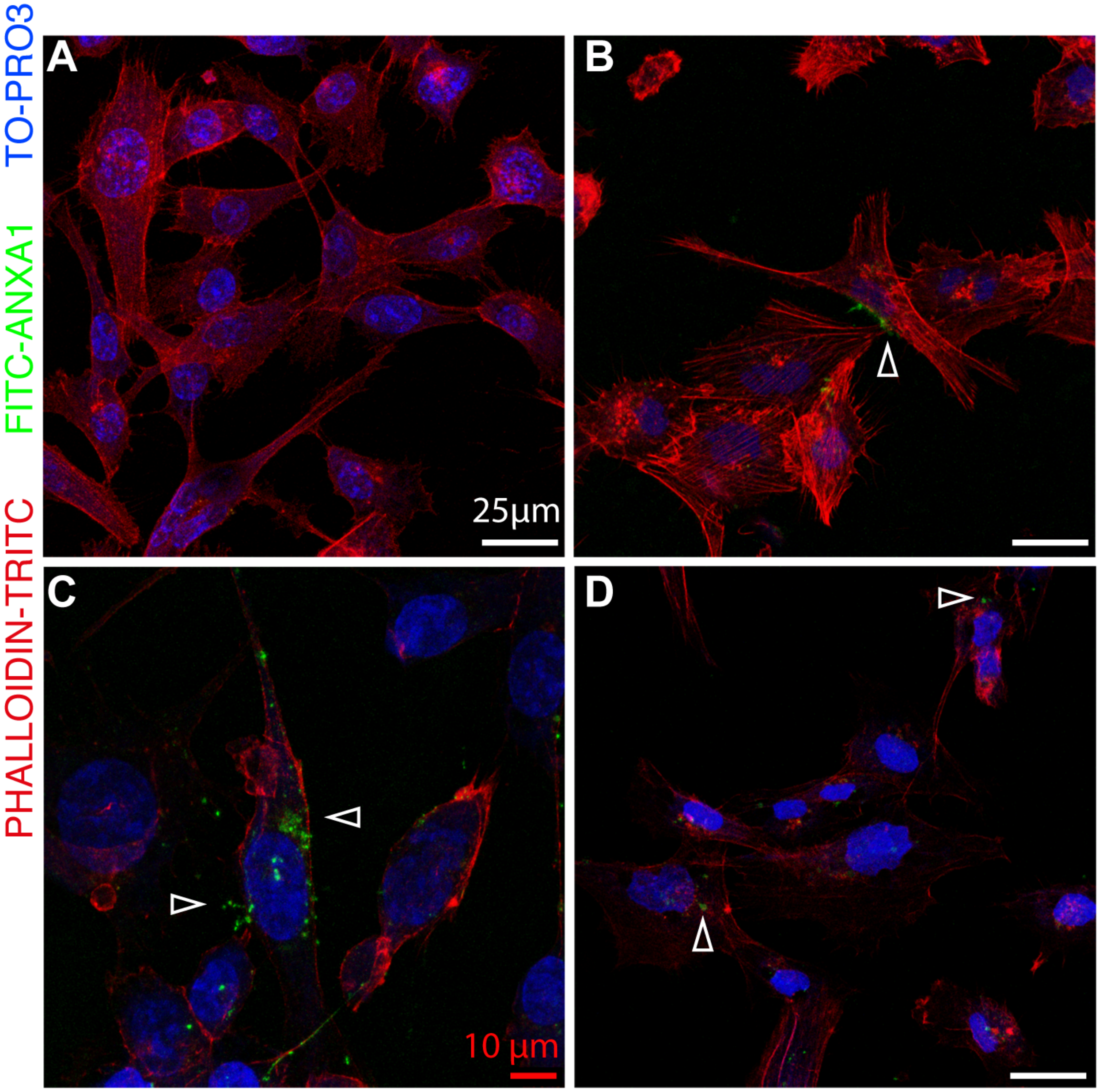

Supplementary Figure 8
