# Supplementary material for: Annexin A1 restores cerebrovascular integrity concomitant with reduced amyloid-β and tau pathology
Source: Brain. 2021 Jun 21;144(5):1526–41. doi: 10.1093/brain/awab050 (PMC8262982; doi:10.1093/brain/awab050)

Annexin-A1 restores cerebrovascular integrity concomitant with reduced amyloid-β and tau pathology

Ries et al.

BRAIN-2020-01457

Supplementary Material – Full-length Western blots

Figure 3C
Left panel
Occludin

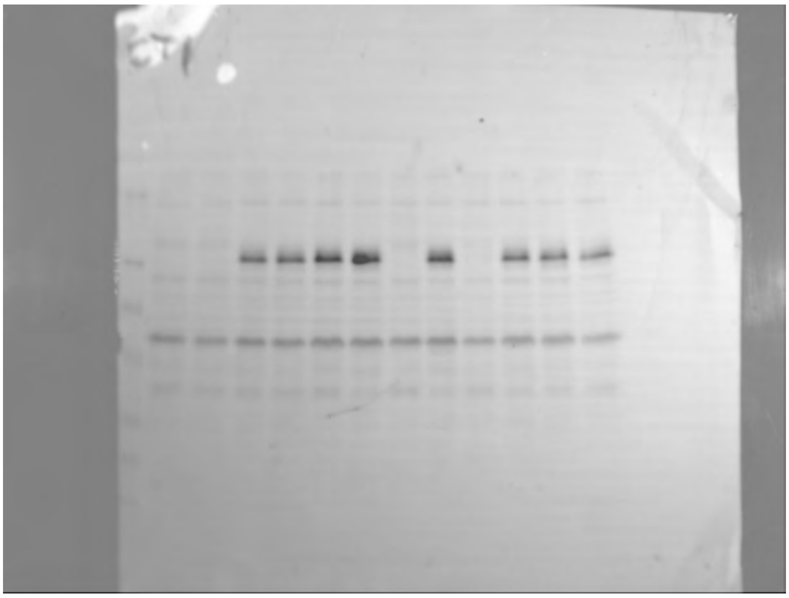

Beta-actin

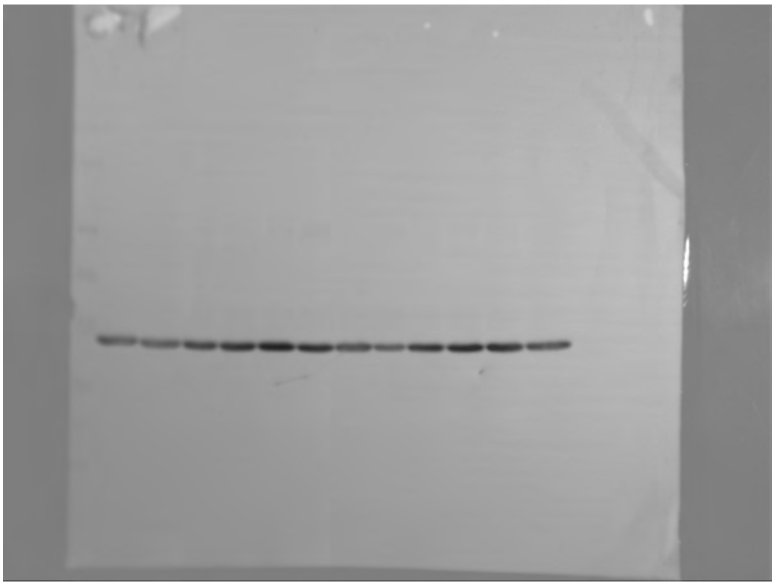

Figure 3C
Right panel
VE-cadherin

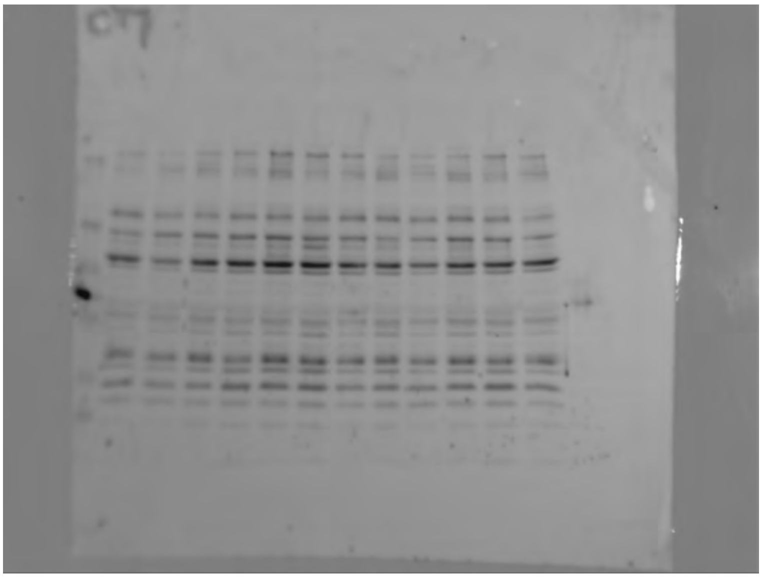

Beta-actin

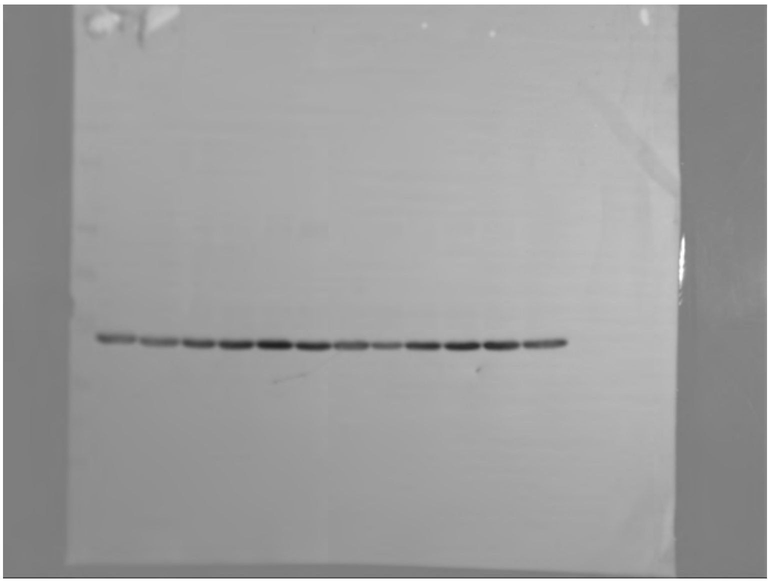

Figure 4F
flAPP

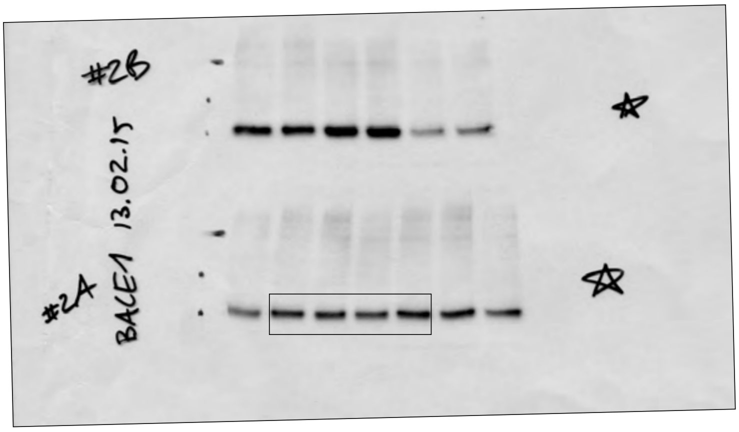

Beta-actin

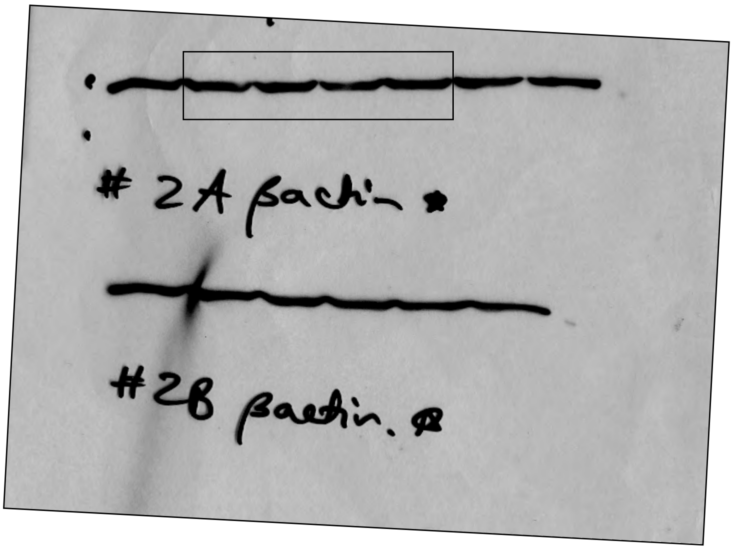

Figure 4F

flAPP

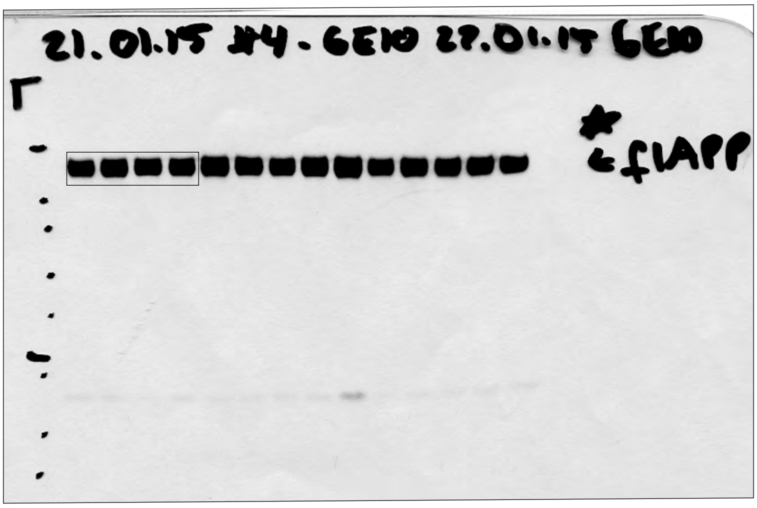

Beta-CTFs

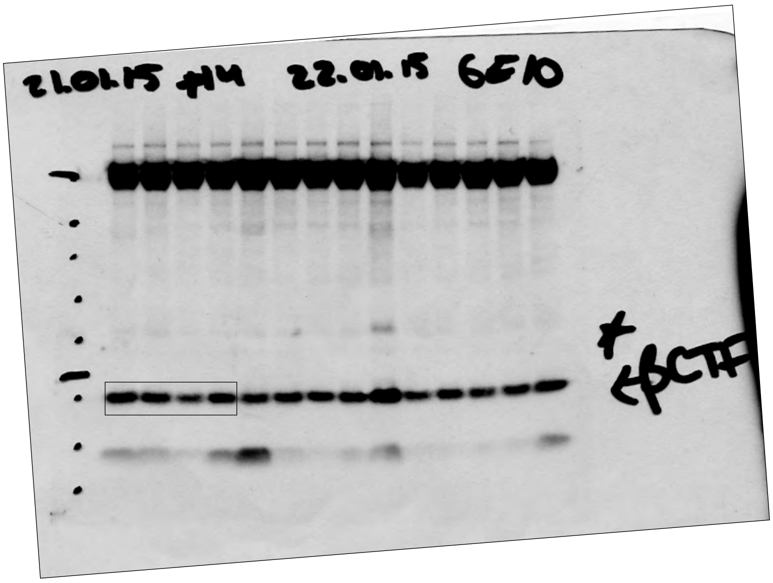

GAPDH

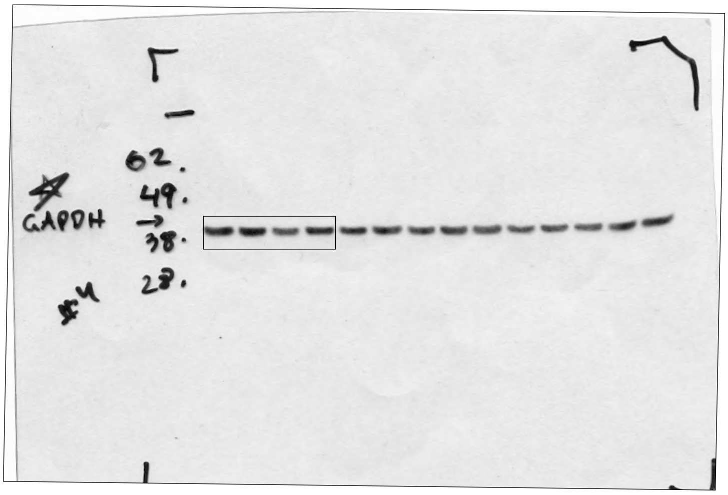

Figure 4G

NEP

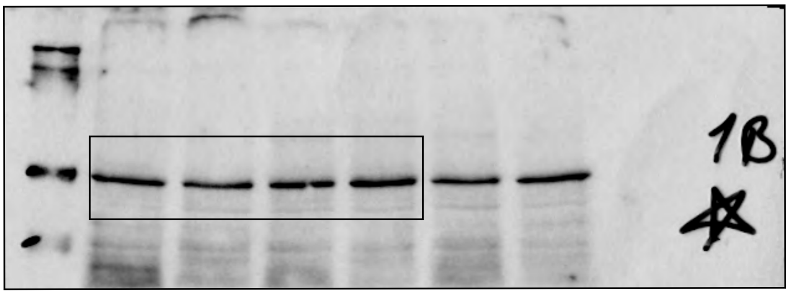

IDE

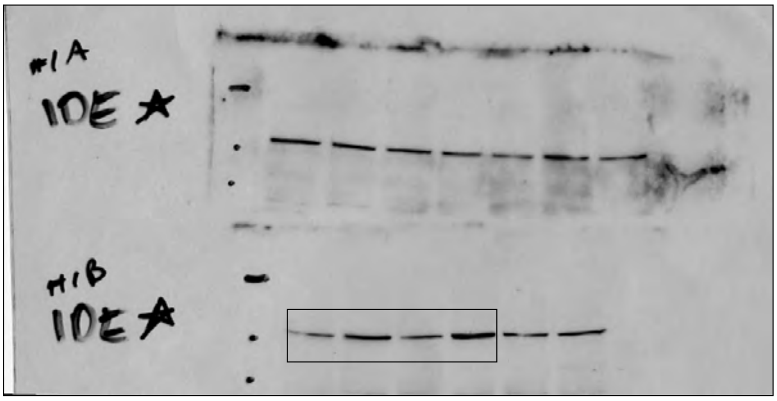

GAPDH

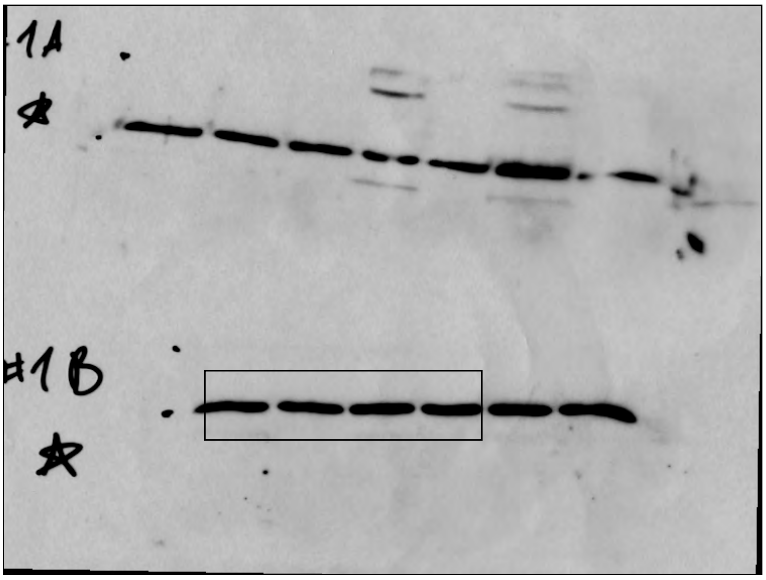

Figure 7E
AT8

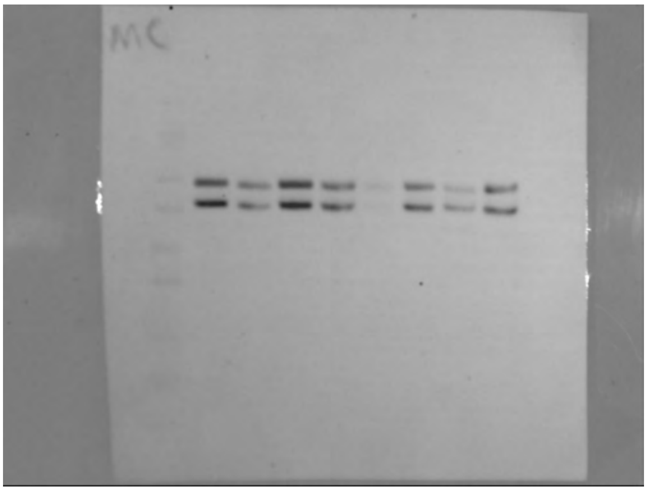

Beta-actin for AT8 blot

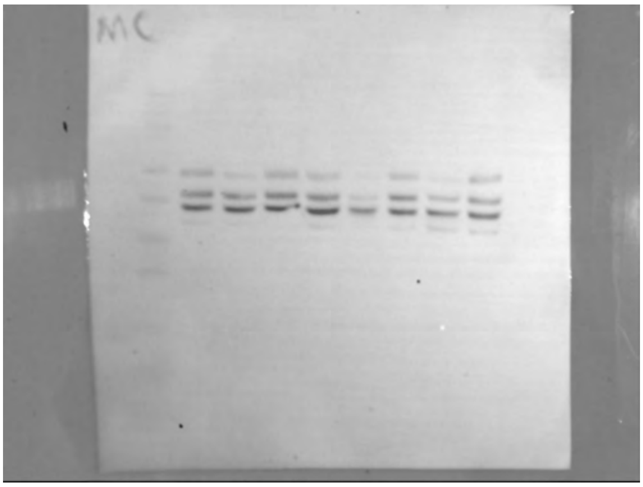

Note : due to the signal strength and proximity of the previous bands to the beta-actin band, the blot was re-run with the same samples and volumes and blotted for beta-actin (see below). The below blot was used for normalisation and is shown in the figure.

Beta-actin re-run for normalisation

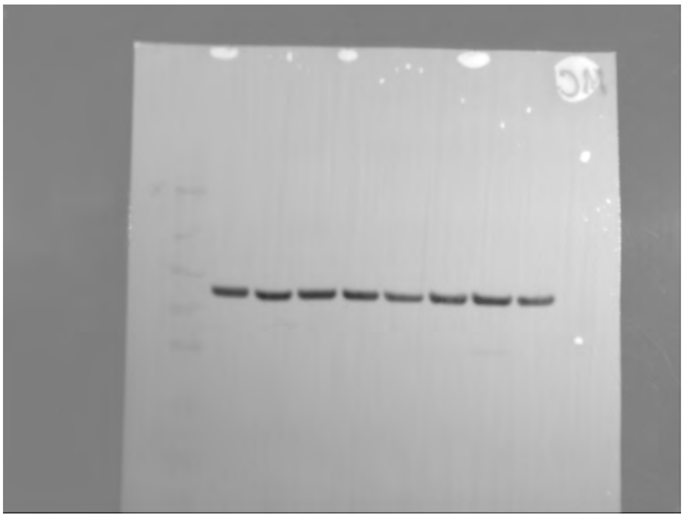

Figure 7F
Tau

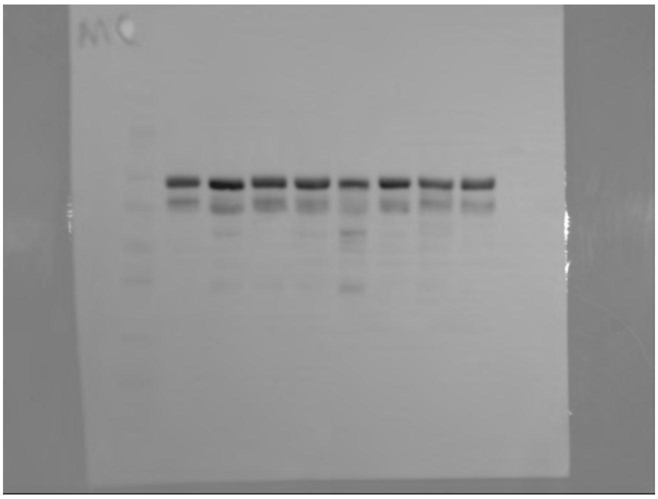

Beta-actin for tau blot

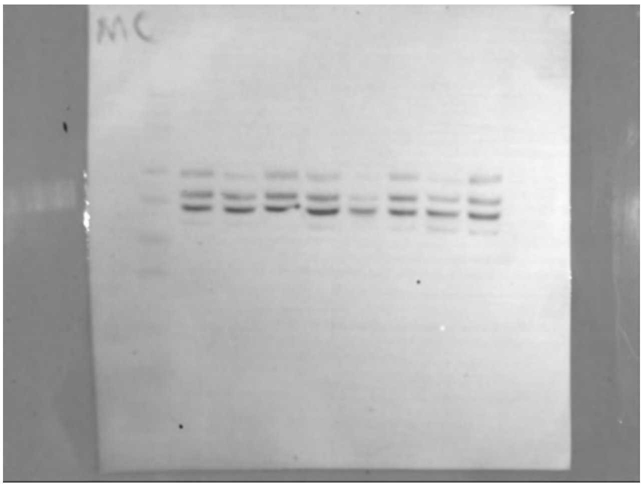

Note : due to the signal strength and proximity of the previous bands to the beta-actin band, the blot was re-run with the same samples and volumes and blotted for beta-actin (see below). The below blot was used for normalisation and is shown in the figure.

Beta-actin – rerun for normalisaion

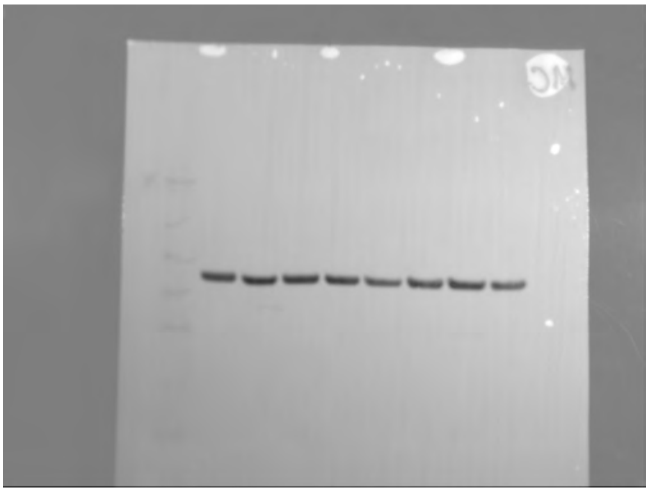

Supplementary figure 1B

AQP4

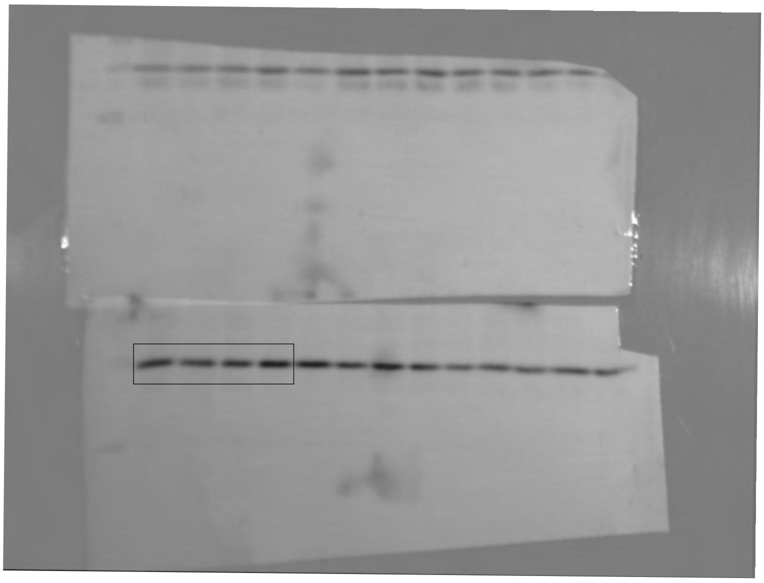

Beta-actin

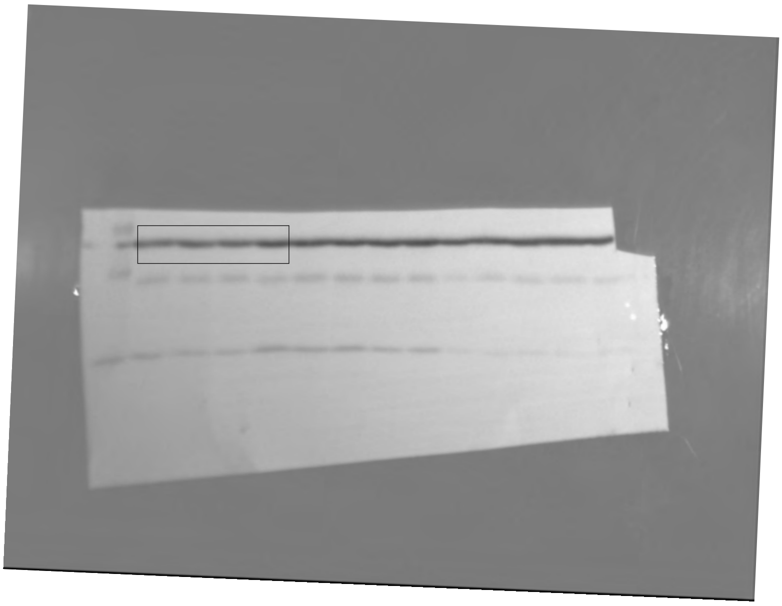

Supplementary Figure 2A
Top panel
BACE1

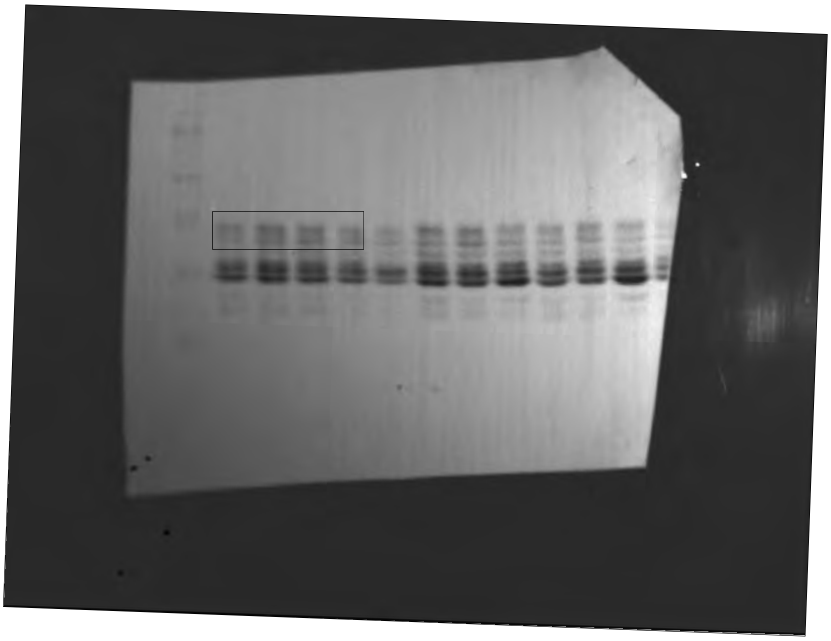

Beta-actin

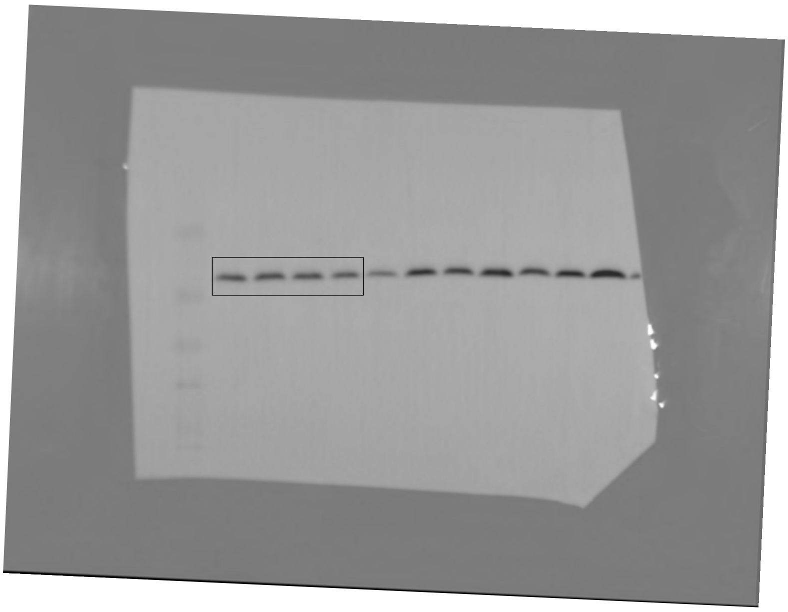

Supplementary Figure 2A
Bottom panel
flAPP

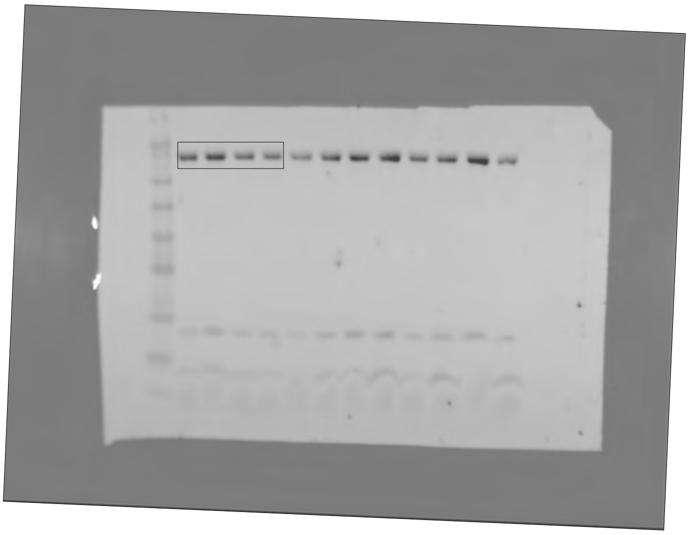

Beta-CTFs

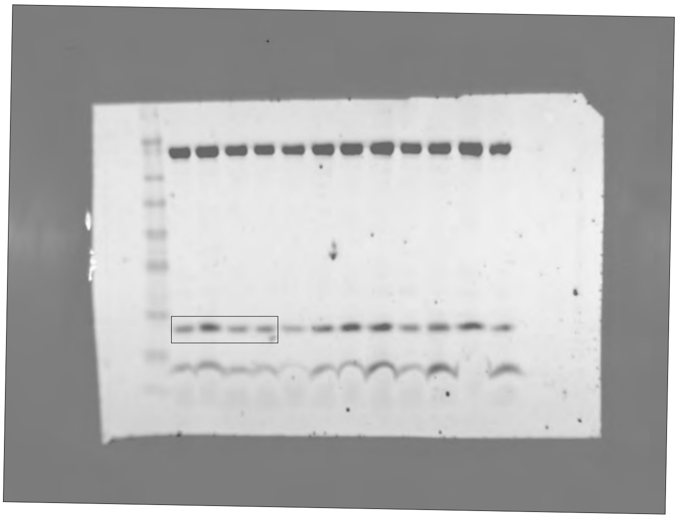

Beta-actin

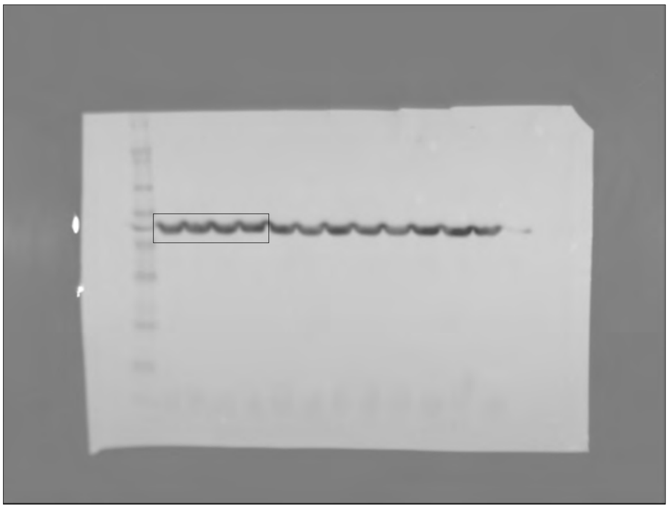

Supplementary Figure 2B
NEP

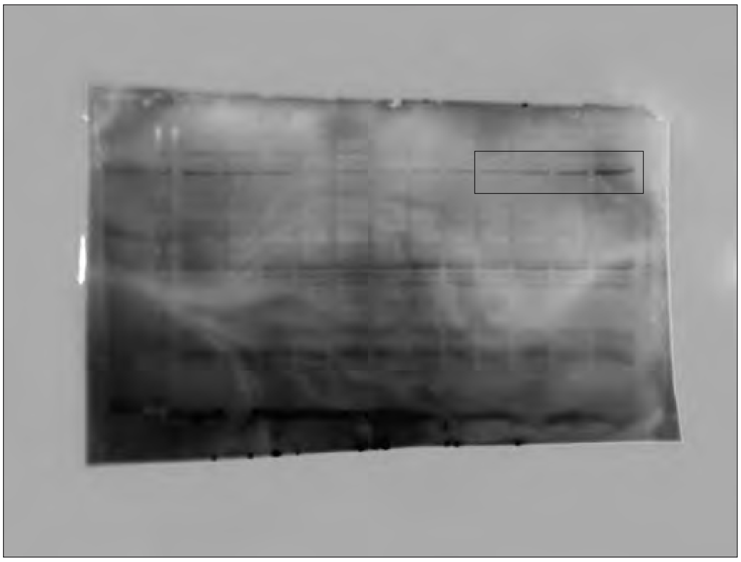

Beta-actin

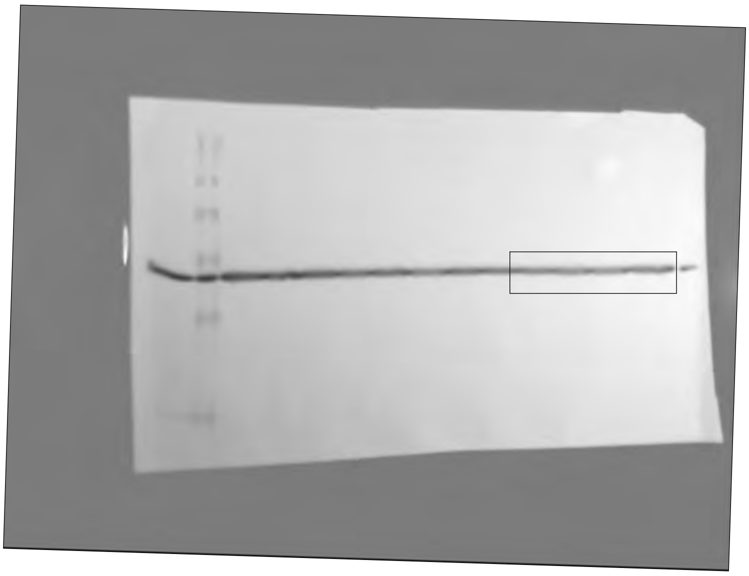

Supplementary Figure 2C
IDE
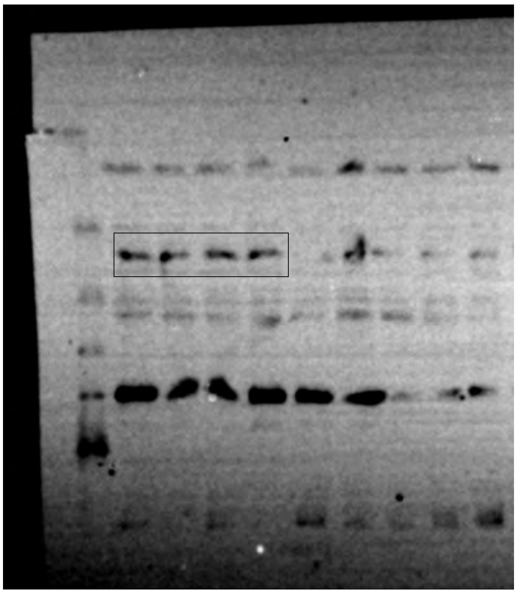

GAPDH
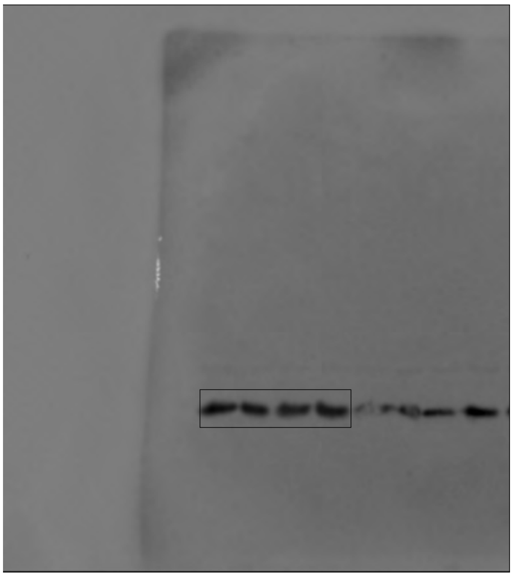

Supplementary Figure 3D
Top panel
LRP1

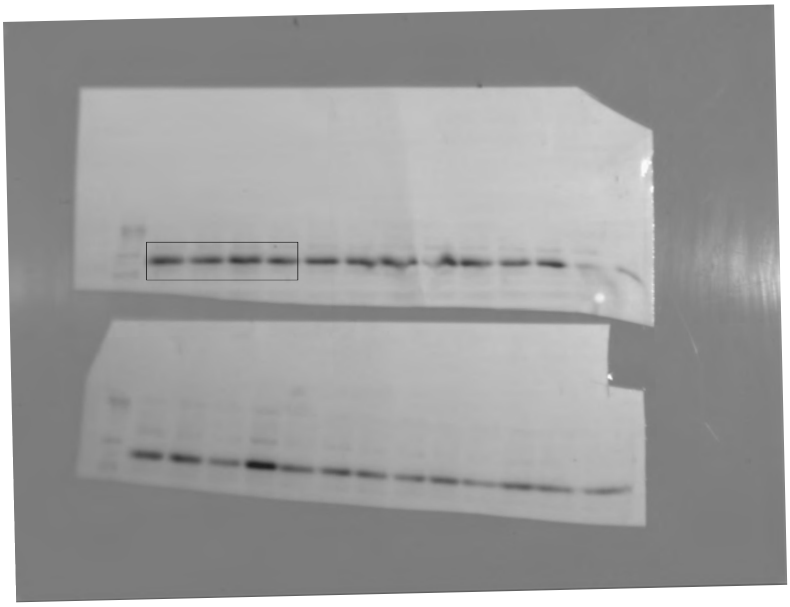

Beta-actin

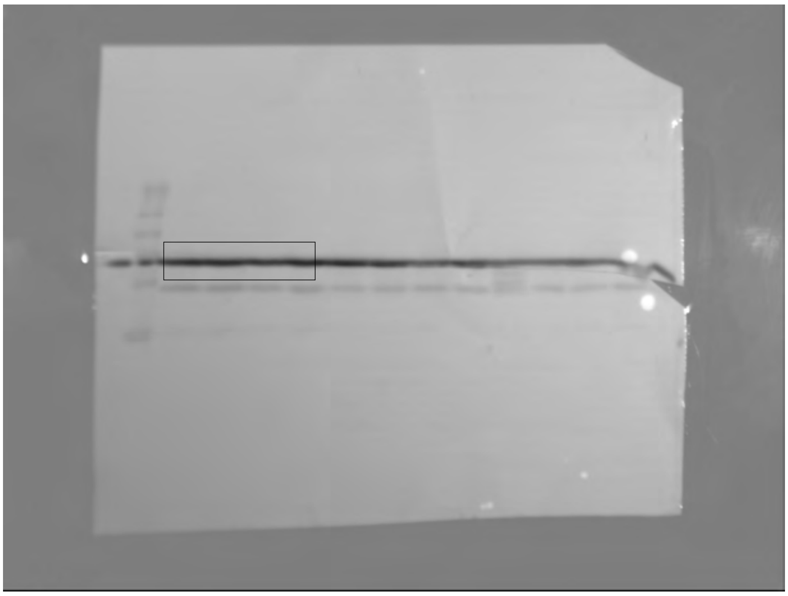

Supplementary Figure 3D
Bottom panel
LRP1

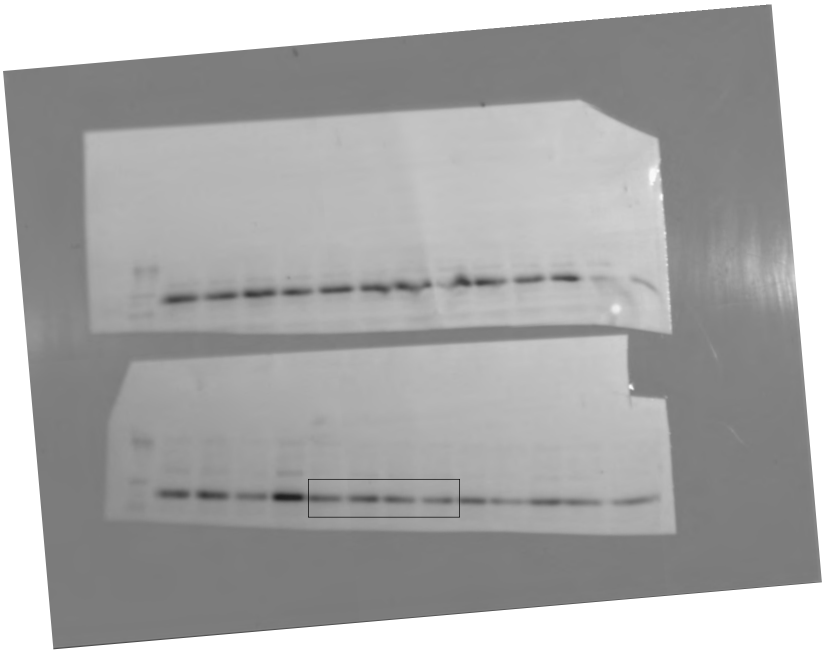

Beta-actin

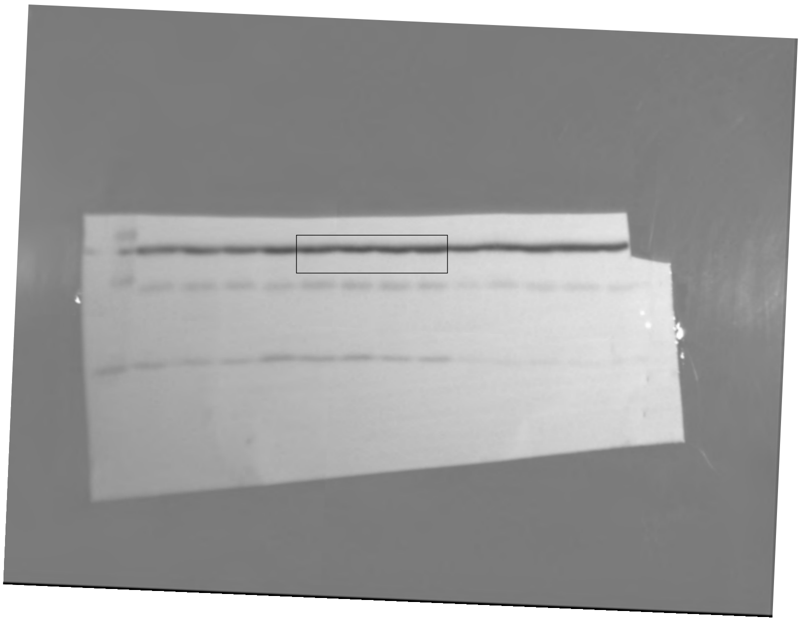

Supplementary Figure 3C
Top panel
BACE1

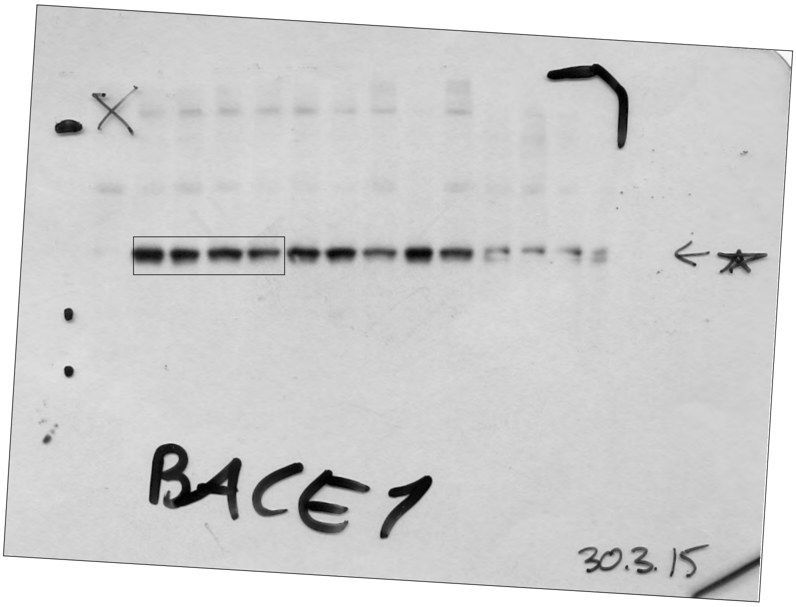

Beta-actin

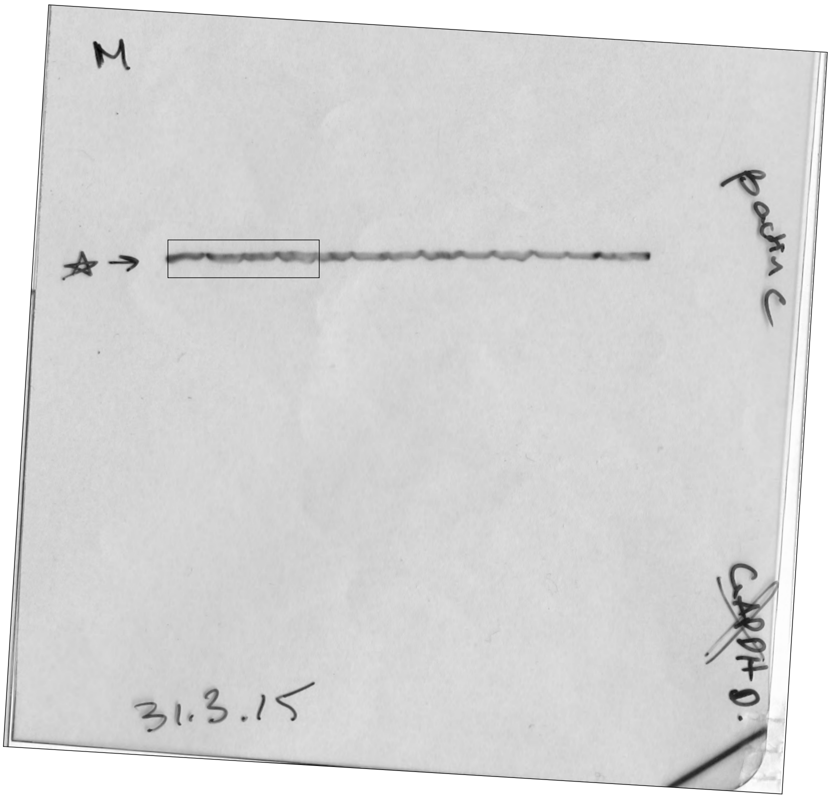

Supplementary Figure 3C
Bottom panel
flAPPs

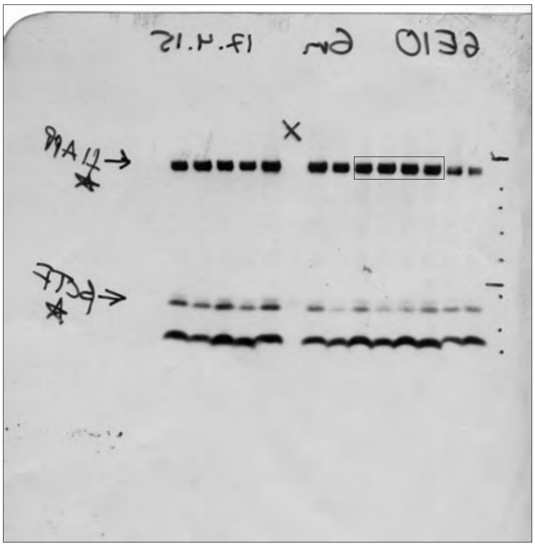

Beta-CTFs

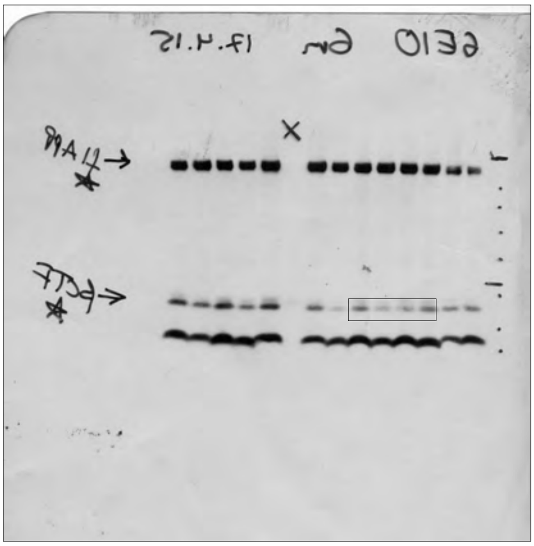

Beta-actin

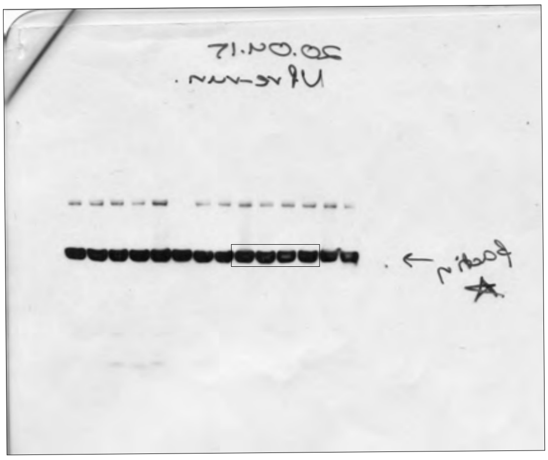

Supplementary figure 3D

NEP

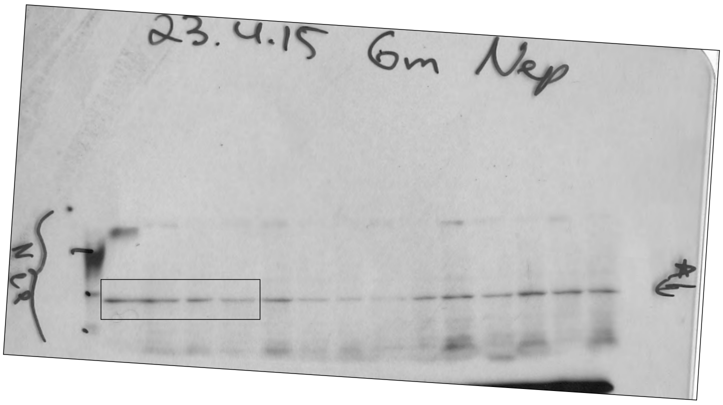

IDE

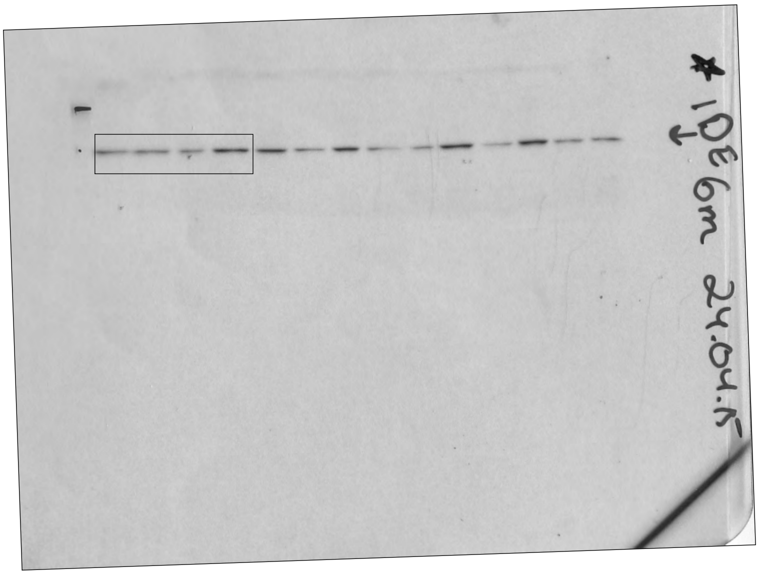

Beta-actin

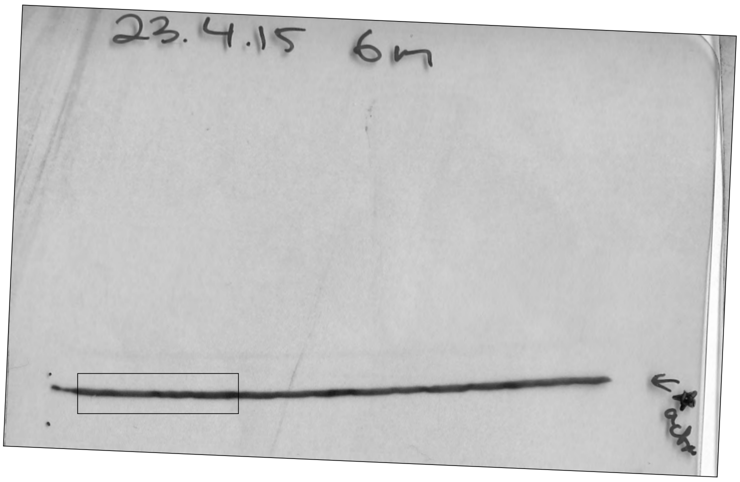

Supplementary figure 4B
Top panel
IBA1

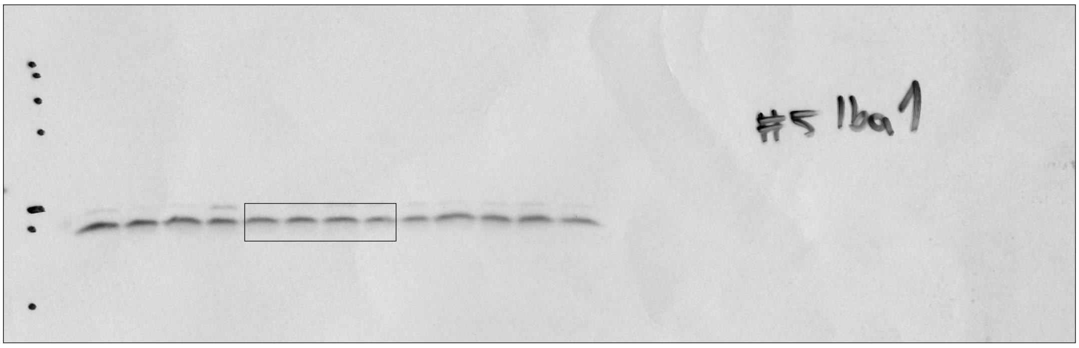

GAPDH

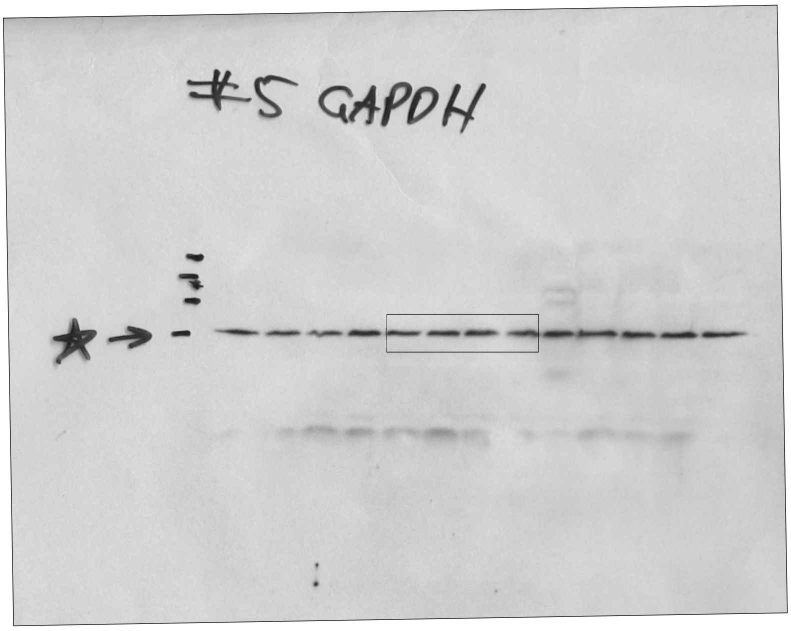

Supplementary figure 4B
Bottom panel
IBA1

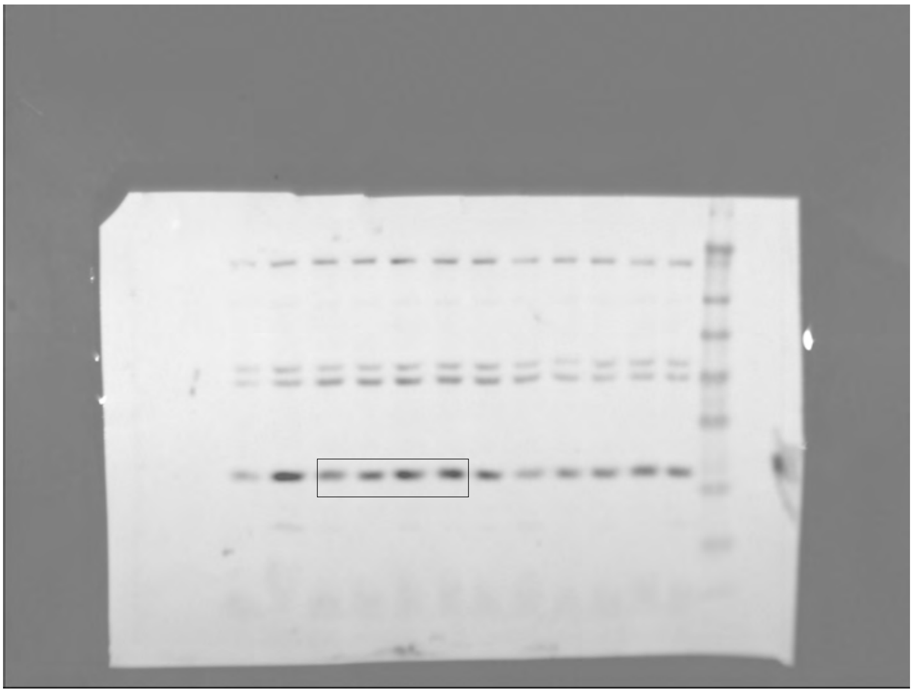

Beta-actin

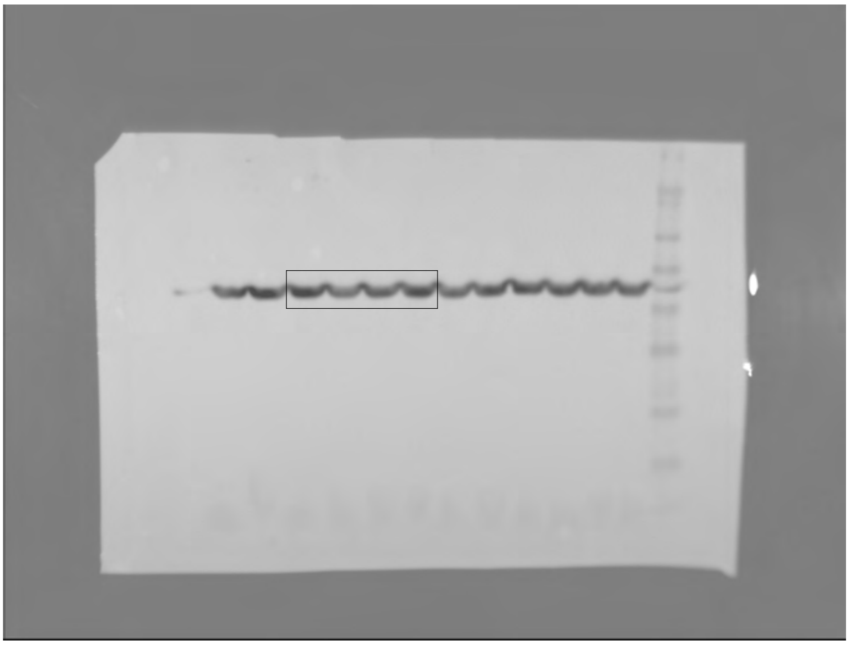

Supplementary figure 4C
Top panel
GFAP

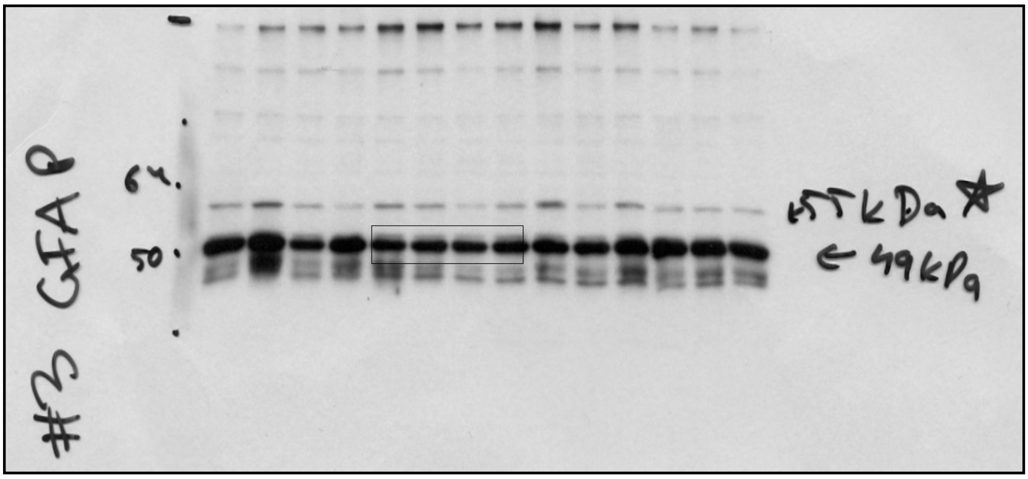

Beta-actin

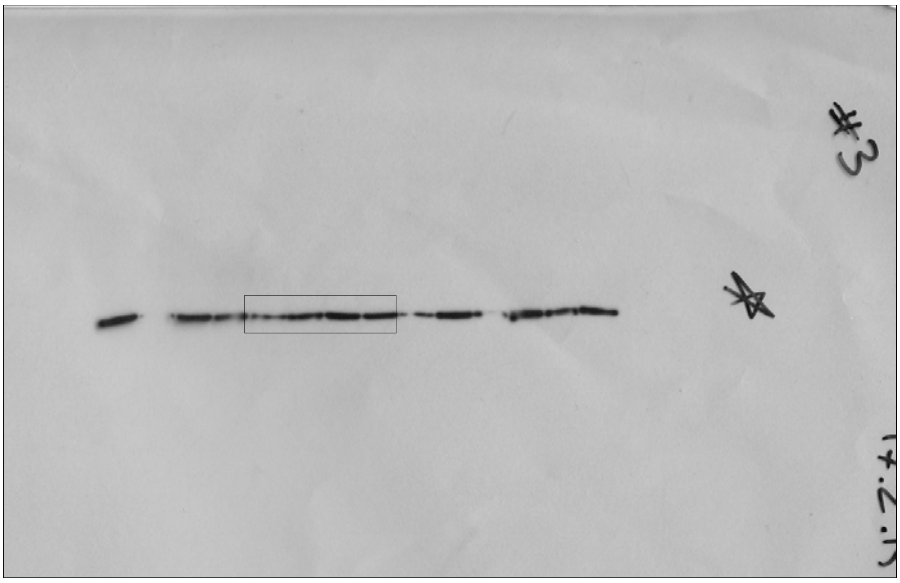

Supplementary figure 4C
Bottom panel
GFAP

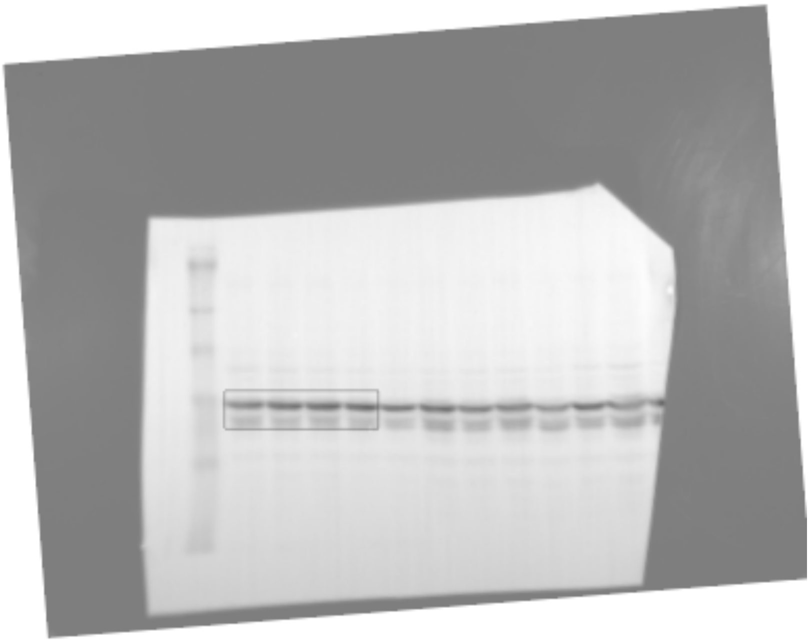

Beta-actin

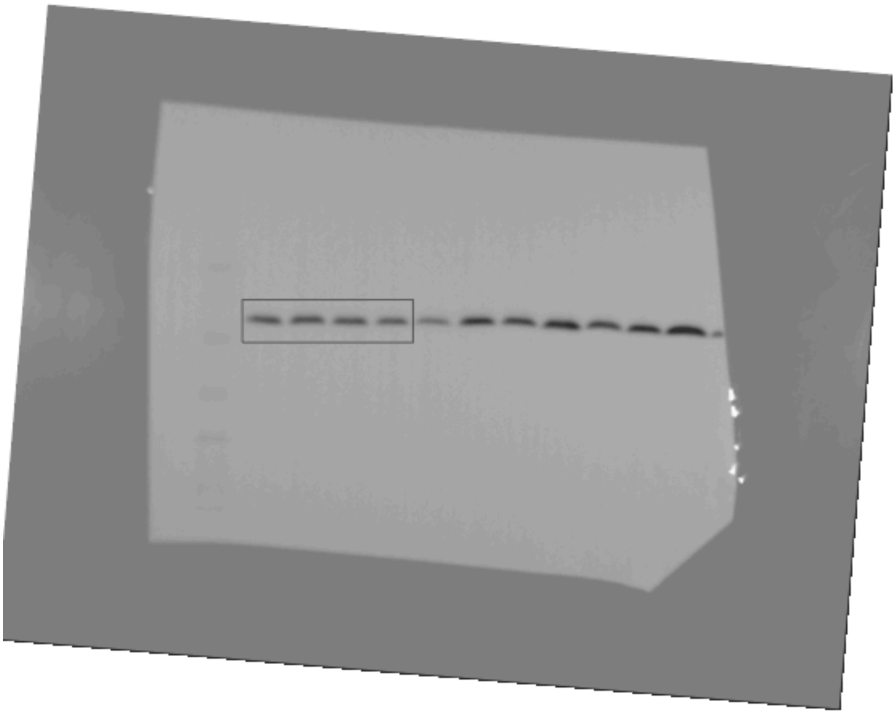

Supplementary figure 6
WT
CTX
Synaptophysin

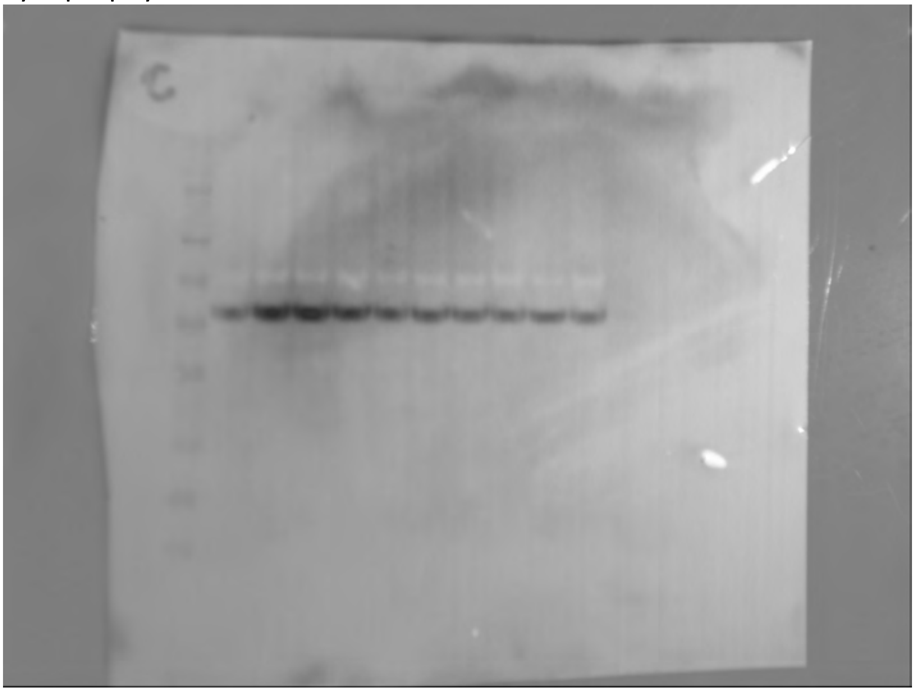

Beta-actin

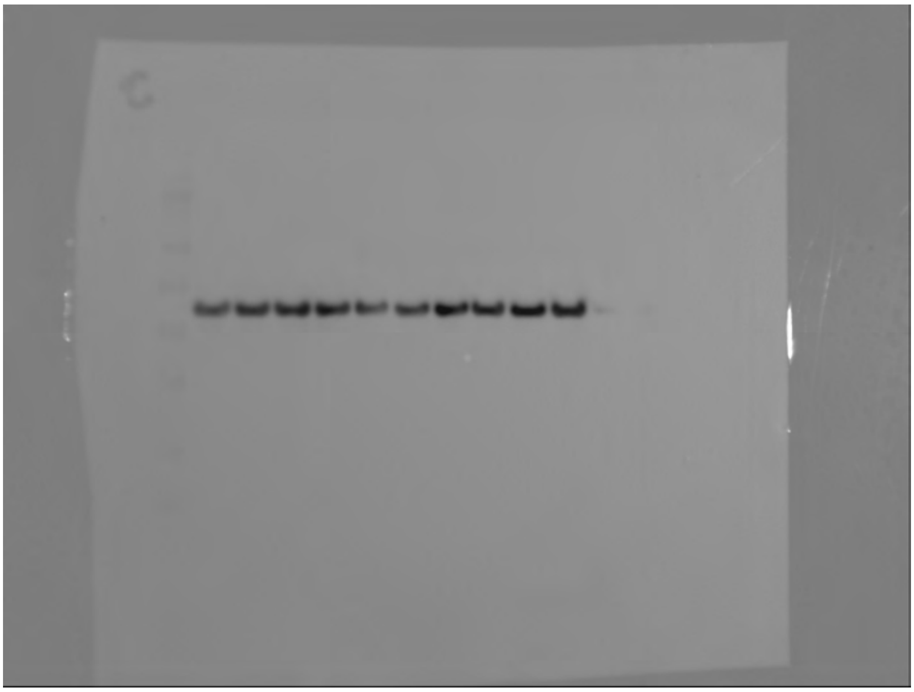

Supplementary figure 6
WT
HC
Synaptophysin

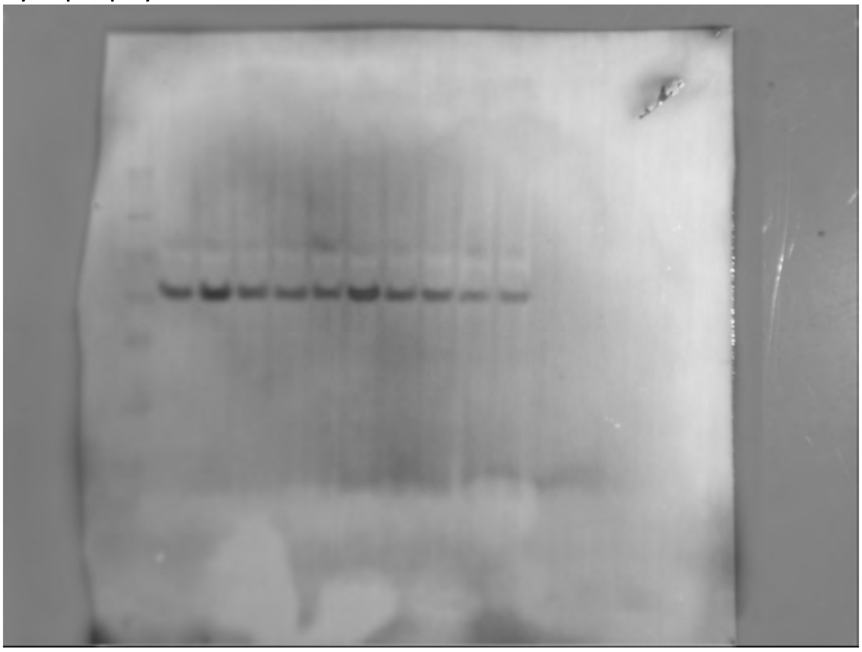

Beta actin

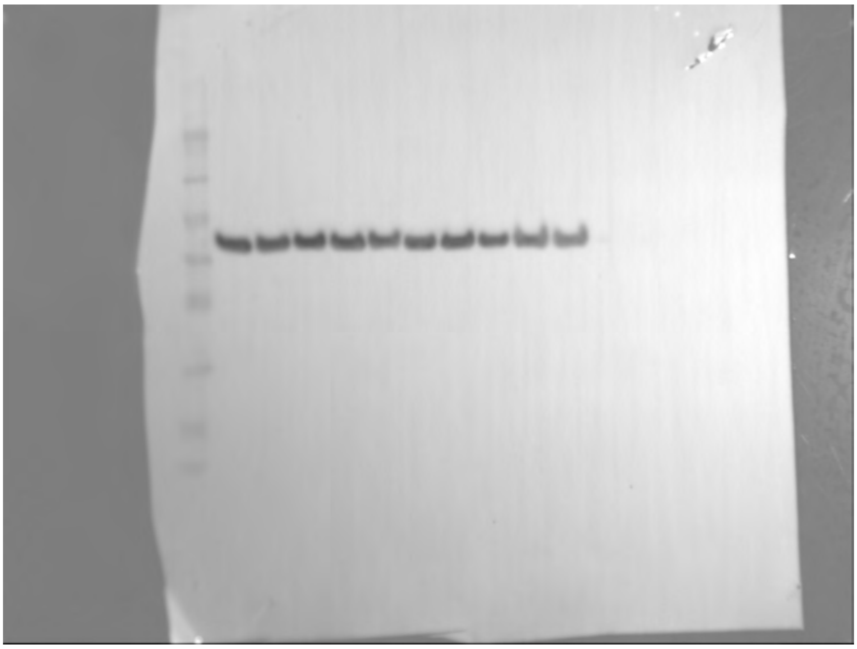

Supplementary figure 6
Tau-P301S
CTX
Synaptophysin

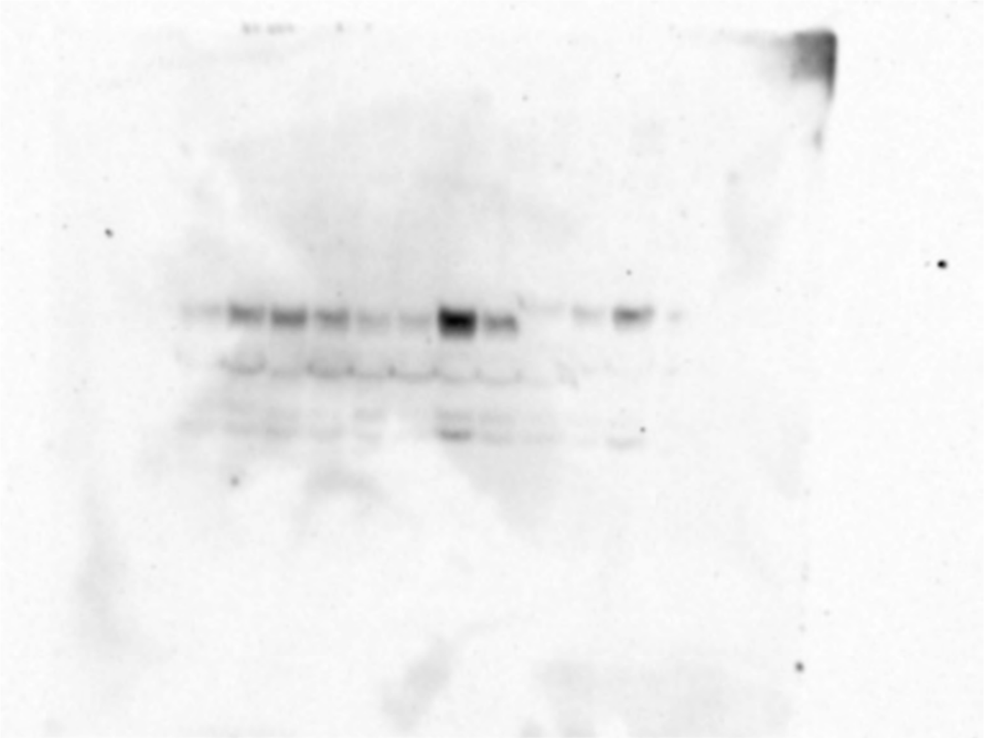

Beta-actin

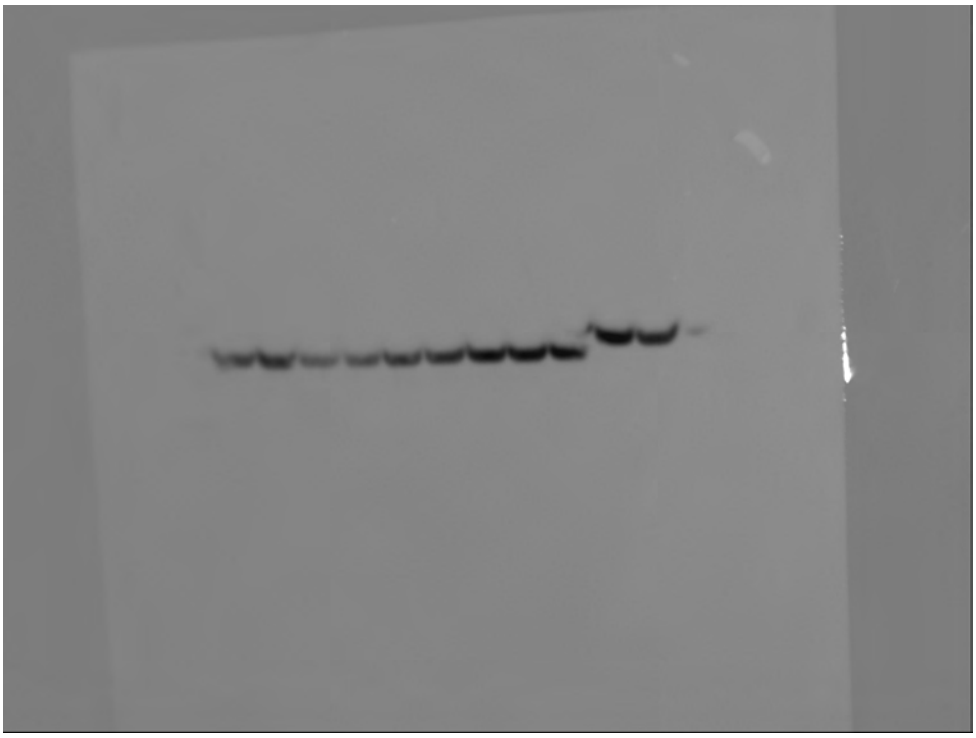

Supplementary figure 6
Tau-P301S
HC
Synaptophysin

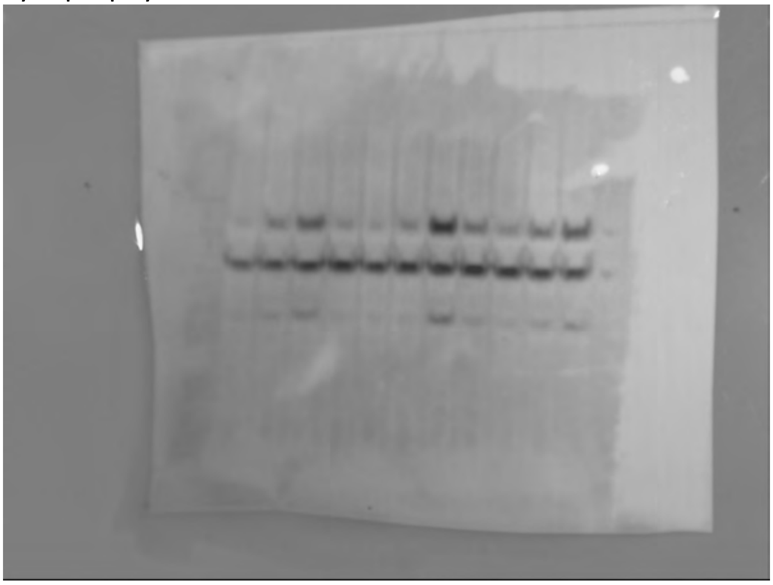

Beta-actin

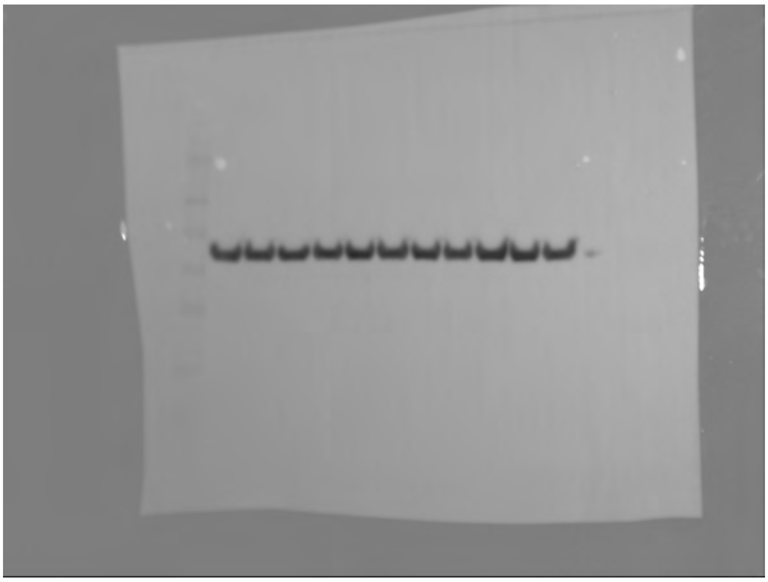

Supplement: awab050_Supplementary_Data [file awab050_supplementary_data.zip › awab050-suppl_data/brain-2020-01457-File010.pdf]
